# Supplementary material for: Integrated transcriptome and metabolome analysis of melanin-related genes and metabolites in Muchuan Black Bones Chickens
Source: PLoS One. 2025 Dec 30;20(12):e0338827. doi: 10.1371/journal.pone.0338827 (PMC12752978; doi:10.1371/journal.pone.0338827)
Supplement: Attachment — Submitted filename: PONE-D-25-36595.pdf [file pone.0338827.s001.pdf]

PLOS ONE

Transcriptome and Metabolome Combined to Analyze the Key Genes and Metabolites Related to the Melanin Content of Muchuan Black Bones Chickens

--Manuscript Draft--

**Manuscript Number:**	PONE-D-25-36595
**Article Type:**	Research Article
**Full Title:**	Transcriptome and Metabolome Combined to Analyze the Key Genes and Metabolites Related to the Melanin Content of Muchuan Black Bones Chickens
**Short Title:**	Melanin-related genes and metabolites were identified in Muchuan Black-Bone Chickens.
**Corresponding Author:**	YE Fei<br>Foshan University<br>Foshan, CHINA
**Keywords:**	Muchuan Black Bones Chickens; transcriptome; metabolome; melanin; CREB5; GIPC2
**Abstract:**	The Muchuan Black Bones Chickens represent a significant subtype of the Sichuan mountain black bones chickens. In the breeding of black bones chickens, the degree of melanization tends to separate due to hybridization, while molecular assisted breeding techniques are still limited, and the key genes involved in melanin deposition in early growth of black bones chickens have yet to be validated. This study aims to identify key genes and metabolites associated with melanin in Muchuan Black Bones Chickens through integrated transcriptomic and metabolomic analyses. CDP-ethanolamine, m-trigallic acid, UDP-4-dehydro-6-deoxy-D-glucose, and glucoiberverin and uridine diphosphate glucuronic acid (UDP glucuronic acid) were identified as significant differential metabolites, which were intimately linked to phenylalanine, tyrosine, and tryptophan biosynthesis associated with melanin accumulation. Total 73 differential expressed genes, including CAMP response element-binding protein 5 (CREB5) and GIPC PDZ domain containing family member 2 (GIPC2), which were enriched in the neuroactive ligand-receptor interaction pathway, arginine-proline metabolism pathway, histidine metabolism, terpenoid backbone biosynthesis, and glycerophospholipid metabolism. This study uncovered important metabolites and regulatory genes related to melanin synthesis, providing a theoretical framework for the breeding and selection of chickens.
**Order of Authors:**	LIU Wei
	JIE Hong-wei
	GAO Ming-chao
	YAO Xiu-mei
	YIN Qiong
	XIANG Hai
	MA Zheng
	ZHANG Zheng-fen
	LI Liang
	LI Hua
	QI Hao
	YE Fei
**Additional Information:**	
**Question**	**Response**
**Financial Disclosure**	Yes

Powered by Editorial Manager® and ProduXion Manager® from Aries Systems Corporation

Enter a financial disclosure statement that describes the sources of funding for the work included in this submission. Review the submission guidelines for detailed requirements. View published research articles from *PLOS ONE* for specific examples.<br><br>This statement is required for submission and **will appear in the published article** if the submission is accepted. Please make sure it is accurate.<br><br>**Funded studies**<br>Enter a statement with the following details:<br>• Initials of the authors who received each award<br>• Grant numbers awarded to each author<br>• The full name of each funder<br>• URL of each funder website<br>• Did the sponsors or funders play any role in the study design, data collection and analysis, decision to publish, or preparation of the manuscript?<br><br>Did you receive funding for this work?	
Please add funding details.<br>as follow-up to "**Financial Disclosure**<br><br>Enter a financial disclosure statement that describes the sources of funding for the work included in this submission. Review the submission guidelines for detailed requirements. View published research articles from *PLOS ONE* for specific examples.<br><br>This statement is required for submission and **will appear in the published article** if the submission is accepted. Please make sure it is accurate.	This research was funded by the Guangdong Basic and Applied Basic Research Foundation (2022A1515111078) and Guizhou Science and Technology Key Project ([2022] No. 034).

**Funded studies**<br>Enter a statement with the following details:<br>• Initials of the authors who received each award<br>• Grant numbers awarded to each author<br>• The full name of each funder<br>• URL of each funder website<br>• Did the sponsors or funders play any role in the study design, data collection and analysis, decision to publish, or preparation of the manuscript?<br><br>Did you receive funding for this work?"	
Please select the country of your main research funder (please select carefully as in some cases this is used in fee calculation).<br>as follow-up to "**Financial Disclosure**<br><br>Enter a financial disclosure statement that describes the sources of funding for the work included in this submission. Review the submission guidelines for detailed requirements. View published research articles from *PLOS ONE* for specific examples.<br><br>This statement is required for submission and **will appear in the published article** if the submission is accepted. Please make sure it is accurate.<br><br>**Funded studies**<br>Enter a statement with the following details:<br>• Initials of the authors who received each award<br>• Grant numbers awarded to each author<br>• The full name of each funder<br>• URL of each funder website<br>• Did the sponsors or funders play any role in the study design, data collection and analysis, decision to publish, or preparation of the manuscript?<br><br>Did you receive funding for this work?"	CHINA - CN
**Competing Interests**	NO authors have competing interests

Use the instructions below to enter a competing interest statement for this submission. On behalf of all authors, disclose any competing interests that could be perceived to bias this work—acknowledging all financial support and any other relevant financial or non-financial competing interests.<br><br>This statement is required for submission and **will appear in the published article** if the submission is accepted. Please make sure it is accurate and that any funding sources listed in your Funding Information later in the submission form are also declared in your Financial Disclosure statement.<br><br>View published research articles from *PLOS ONE* for specific examples.<br><br>**NO authors have competing interests**<br><br>Enter: *The authors have declared that no competing interests exist.*<br><br>**Authors with competing interests**<br><br>Enter competing interest details beginning with this statement:<br><br>*I have read the journal's policy and the authors of this manuscript have the following competing interests: [insert competing interests here]*<br><br>* typeset	Enter: The authors have declared that no competing interests exist.
**Ethics Statement**<br><br>Enter an ethics statement for this submission. This statement is required if the study involved:<br><br>• Human participants<br>• Human specimens or tissue<br>• Vertebrate animals or cephalopods<br>• Vertebrate embryos or tissues	Vertebrate animals or cephalopods,FOSU#20210031-01

• Field research<br><br>Write "N/A" if the submission does not require an ethics statement.<br><br>General guidance is provided below. Consult the submission guidelines for detailed instructions. **Make sure that all information entered here is included in the Methods section of the manuscript.**	

Powered by Editorial Manager® and ProduXion Manager® from Aries Systems Corporation

**Format for specific study types**<br><br>**Human Subject Research (involving human participants and/or tissue)**<br>• Give the name of the institutional review board or ethics committee that approved the study<br>• Include the approval number and/or a statement indicating approval of this research<br>• Indicate the form of consent obtained (written/oral) or the reason that consent was not obtained (e.g. the data were analyzed anonymously)<br><br>**Animal Research (involving vertebrate animals, embryos or tissues)**<br>• Provide the name of the Institutional Animal Care and Use Committee (IACUC) or other relevant ethics board that reviewed the study protocol, and indicate whether they approved this research or granted a formal waiver of ethical approval<br>• Include an approval number if one was obtained<br>• If the study involved *non-human primates*, add *additional details* about animal welfare and steps taken to ameliorate suffering<br>• If anesthesia, euthanasia, or any kind of animal sacrifice is part of the study, include briefly which substances and/or methods were applied<br><br>**Field Research**<br><br>Include the following details if this study involves the collection of plant, animal, or other materials from a natural setting:<br>• Field permit number<br>• Name of the institution or relevant body that granted permission	
**Data Availability**<br><br>Authors are required to make all data underlying the findings described fully available, without restriction, and from the time of publication. PLOS allows rare exceptions to address legal and ethical concerns. See the PLOS Data Policy and FAQ for detailed information.	Yes - all data are fully available without restriction

Powered by Editorial Manager® and ProduXion Manager® from Aries Systems Corporation

A Data Availability Statement describing where the data can be found is required at submission. Your answers to this question constitute the Data Availability Statement and **will be published in the article**, if accepted.<br><br>**Important:** Stating 'data available on request from the author' is not sufficient. If your data are only available upon request, select 'No' for the first question and explain your exceptional situation in the text box.<br><br>Do the authors confirm that all data underlying the findings described in their manuscript are fully available without restriction?	
**Describe where the data may be found in full sentences. If you are copying our sample text, replace any instances of XXX with the appropriate details.**<br><br>• If the data are **held or will be held in a public repository**, include URLs, accession numbers or DOIs. If this information will only be available after acceptance, indicate this by ticking the box below. For example: *All XXX files are available from the XXX database (accession number(s) XXX, XXX.).*<br>• If the data are all contained **within the manuscript and/or Supporting Information files**, enter the following: *All relevant data are within the manuscript and its Supporting Information files.*<br>• If neither of these applies but you are able to provide **details of access elsewhere**, with or without limitations, please do so. For example:<br><br>*Data cannot be shared publicly because of [XXX]. Data are available from the XXX Institutional Data Access / Ethics Committee (contact via XXX) for researchers who meet the criteria for access to confidential data.*<br><br>*The data underlying the results presented in the study are available from (include the name of the third party*	All Transcriptome and metabolome files are available from the CNGB Sequence Archive (CNSA) of China National GeneBank DataBase (CNGBdb) with accession number CNP0006904.

Powered by Editorial Manager® and ProduXion Manager® from Aries Systems Corporation

and contact information or URL).<br>• This text is appropriate if the data are owned by a third party and authors do not have permission to share the data.<br><br>* typeset	
Additional data availability information:	Tick here if the URLs/accession numbers/DOIs will be available only after acceptance of the manuscript for publication so that we can ensure their inclusion before publication.

Powered by Editorial Manager® and ProduXion Manager® from Aries Systems Corporation

Manuscript

Click here to access/download;Manuscript;Manuscript---LIU Wei-7.9-1.docx

Transcriptome and Metabolome Combined to Analyze the Key Genes and Metabolites Related to the Melanin Content of Muchuan Black Bones Chickens

LIU Wei[1], JIE Hong-wei[1], GAO Ming-chao[2], YAO Xiu-mei[1], YIN Qiong[1], XIANG Hai[1], MA Zheng[1], ZHANG Zheng-fen[2], LI Liang[3], LI Hua[2], QI Hao[1*], YE Fei[1*]

1 *Guangdong Provincial Key Laboratory of Animal Molecular Design and Precise Breeding, School of Animal Science and Technology, Foshan University, Foshan,528225, China;*

2 *Guangdong Tinoo's Food Co., Ltd., Qingyuan, 511500, China*

3 *Animal Husbandry and Veterinary Institute of Guizhou Province, Guizhou, 550005, China*

[*] Correspondence: QI Hao: q15663857127@163.com;

YE Fei, e-mail:yefei0831@fosu.edu.cn

Simple Summary: Through the integration of transcriptomics and metabolomics, the key genes and metabolites of Muchuan Black Bones Chickens were comprehensively explored to deepen the understanding of melanin formation. *CAMP response element-binding protein 5* (*CREB5*) and *GIPC PDZ domain containing family member 2* (*GIPC2)*, related to melanin content, were identified through this transcriptome analysis. In the differential metabolite analysis, this study identified Glucose-6-phosphate and UDP-glucuronic acid as key metabolites that were significantly associated with melanin deposition, which indirectly affected the formation of melanin.

Abstract: The Muchuan Black Bones Chickens represent a significant subtype of the Sichuan mountain black bones chickens. In the breeding of black bones chickens, the degree of melanization tends to separate due to hybridization, while molecular assisted breeding techniques are still limited, and the key genes involved in melanin deposition in early growth of black bones chickens have yet to be validated. This study aims to identify key genes and metabolites associated with melanin in Muchuan Black Bones Chickens through integrated transcriptomic and metabolomic analyses. CDP-ethanolamine, m-trigallic acid, UDP-4-dehydro-6-deoxy-D-glucose, and glucoiberverin and uridine diphosphate glucuronic acid (UDP glucuronic acid) were identified as significant differential metabolites, which were intimately linked to phenylalanine, tyrosine, and tryptophan biosynthesis associated with melanin accumulation. Total 73 differential expressed genes, including *CAMP response element-binding protein 5 (CREB5) and GIPC PDZ domain containing family*

member 2 (GIPC2), which were enriched in the neuroactive ligand-receptor interaction pathway, arginine-proline metabolism pathway, histidine metabolism, terpenoid backbone biosynthesis, and glycerophospholipid metabolism. This study uncovered important metabolites and regulatory genes related to melanin synthesis, providing a theoretical framework for the breeding and selection of chickens.

Keywords: Muchuan Black Bones Chickens, transcriptome, metabolome, melanin, *CREB5*， *GIPC2*

1. Introduction

Muchuan Black Bones Chickens, also referred to as "West Sichuan Black Bones Chickens" [1] is renowned for its distinctive black bones and rich nutritional value[2-4]. However, Muchuan Black Bones Chickens exhibit segregation in melanin deposition, which emerged in the early stages of growth. Melanin can be divided into five different types: eumelanin, fusomelanin, isomelanin, neuromelanin, and pyomelanin[5]. Black bone chickens primarily consist of eumelanin, a polymer made from 5, 6-dihydroxyindole (DHI) and 5, 6-dihydroxyindole-2-carboxylic acid (DHICA)[6]. The synthesis and transport of melanin were regulated by multiple genes, such as *Endothelin Receptor Type B2* (*EDNRB2*)[7], *Endothelin 3* (*EDN3*)[8], *tyrosinase* (*TYR*)[9], *tyrosinase-related protein 1 (TYRP1)[10], microphthalmia-associated transcription factor* (*MITF*)[11], and *melanosome protein 17* (*PMEL17*)[12]. *EDNRB2*, a G protein-coupled receptor, activates downstream signaling pathways upon binding with *EDN3,* which supports the migration of neural crest-derived melanoblasts to skin and feather regions. The *TYR* and *TYRP1* families formed an enzymatic network for melanin synthesis, where *TYR* catalyzed the oxidation of tyrosine to dopaquinone, while *TYRP1* stabilized *TYR* enzyme activity, catalyzed the isomerization of dopa pigment, and facilitated deposition onto the melanosome matrix fiber formed by *PMEL17*. The *MLPH* gene dynamically regulated melanin from synthesis to distribution, whereas *MITF* influenced the expression of *MLPH* and indirectly affected melanin formation. Furthermore, the interaction between *MITF* and the M-box motif was critical for the coordinated

expression of tyrosinase family genes, underscoring the regulatory significance of *MITF* in melanin formation. Previously, molecular biological methods for studying melanin deposition included gene expression analysis[13], SNP molecular marker screening[14], transcriptomics, and proteomics[15]. Melanin content was determined using spectrophotometry[16], high-performance liquid chromatography (HPLC)[17], and fluorescence analysis[18], while melanin extraction was achieved through enzymolysis[19], a three-step method (degreasing-enzymolysis-acidolysis)[20].

At the early growth stage of black bones chickens, melanin is deposited in the breast and skin with a marked degree of separation. To identify candidate molecular markers connected to melanin deposition in the early development phase of Muchuan Black Bones Chickens, this study utilized 35-day-old Muchuan Black Bones Chickens as experimental subjects to identify key genetic markers and metabolites associated with early breast muscle melanin deposition.

2. Materials and Methods

2.1 Ethics Statement

The Animal Care Committee at Foshan University (Foshan, People's Republic of China; Approval ID: FOSU#20210031-01) approved the research. All animal-related experiments followed the National and International Guidelines for Animal Welfare.

2.2 Sample Collection

All Muchuan Black Bones Chickens were sourced from Muchuan County Black Phoenix Black Bone Chickens Industry Co., Ltd. The chickens were raised at the Foshan Animal Breeding Engineering Technology Research Center, following

standard broiler feeding protocols until 35 days of age. A total of 60 35-day-old Muchuan Black Bones Chickens were selected.. Euthanasia was carried out by using the gas fainting method，and whole blood (2 ml) was collected via wing veins and stored in collection vessels at -20°C for sequencing purposes. Breast muscle was also collected and stored at -80° C for subsequent analysis.

2.3 Determination of Melanin in Breast Muscle

The melanin concentration in the breast muscles was determined through a chicken melanin ELISA kit (manufacturer: Shanghai Enzyme Biotech Co., Ltd.; model: YJ500392). A total of 0.1 g of breast muscle was homogenized in PBS buffer, and the supernatant was collected. Standard wells, blank control wells, and test sample wells were marked on an enzyme-linked immunosorbent assay (ELISA) plate. Three technical replicates were established for each condition, with corresponding reagents and samples added according to the manufacturer's instructions. Following incubation and washing steps, a termination solution was added, and the OD value (absorbance) of each well was measured at a wavelength of 450 nm using an ELISA reader, with results recorded within 15 minutes[21].

2.4 RNA-seq Sequencing

Based on the melanin content in the breast muscles, the samples were categorized into a high melanin content group (MH) and a low melanin content group (ML). Five chickens from each group were selected, and appropriate amounts of breast muscle tissue samples were collected for mRNA transcriptome sequencing. To guarantee the reliability of transcriptome sequencing data, the extracted total RNA

samples were subjected to rigorous quality control assessments, including purity evaluation, concentration measurement, and integrity verification[22]. The sample concentrations ranged between 420-740 ng/μL, with OD 260/280 values[23] approximately around 2.0, and all integrity evaluation index RIN values exceeding 7 [24]. All total RNAs met quality standards and were suitable for subsequent experimental steps. First, RNA purity and concentration were measured using a NanoDrop 2000 spectrophotometer, followed by RNA integrity detection using the Agient2100/LabChip GX. After the samples passed the test, the library was constructed, including mRNA interruption and enrichment, cDNA purification, end repair, A-tail and splicing connected for sequencing, fragment size selection with AMPure XP beads, and cDNA library obtained by PCR enrichment. After the library construction was completed, the quality control of the library was carried out. After passing quality inspection, the transcriptome library was sequenced in PE150 mode using the Illumina NovaSeq 6000 sequencing platform. After the sequencing data were obtained, BMKCloud (https://international.biocloud.net/zh/dashboard) was used to analyze differentially expressed genes through the DESeq2-edgeR methods, with an FDR threshold of 0.05 and a fold change (FC) of 1.5 as the criterion for significance. Differentially expressed genes were analyzed for functional enrichment using the DAVID Bioinformatics Resources database (https://david.ncifcrf.gov/), including pathway enrichment analyses from the Gene Ontology (GO) and the Kyoto Encyclopedia of Genes and Genomes (KEGG). Additionally, Sangerbox 3.0 (http://www.sangerbox.com/) was used to visualize genetic variations through volcano

plots and to generate GO and KEGG bubble charts.

2.5 Assessment via Quantitative Real-Time PCR (qPCR)

Reverse transcription was performed with HiScript III All-in-one RT SuperMix (Vazyme, China), yielding cDNA at concentrations ≥1 ng/μL. Primer sequences were designed using the NCBI's Primer-BLAST platform. Quantitative PCR was carried out with SYBR Green Master Mix (Vazyme) to determine relative mRNA expression. The Qubit 3.0 （Thermo）fluorometer was used. The results are analyzed based on calculation 2 $^{\Delta\Delta}$Ct. The quality assessment of the constructed library was conducted using the Qsep400 high-throughput analysis system. Subsequently, paired-end sequencing (PE150) was performed on the Illumina NovaSeq6000 sequencing platform.

2.6 Extraction and Detection of Metabolites

Metabolomic analysis was performed through a standardized protocol involving initial metabolite extraction, instrumental detection, and final metabolite identification and quantification. Firstly, six chickens in the ML group and six chickens in the MH group were selected for metabolite extraction. The metabolite extraction mainly includes the following steps: following the addition of extraction solvent and magnetic beads, samples underwent mechanical grinding, ultrasonic treatment, static centrifugation, and subsequent vacuum drying of the supernatant.[25]. Subsequently, the reconstituted extracts were analyzed using an integrated UPLC-QTOF-MS system (Waters Acquity I-Class PLUS coupled with Xevo G2-XS). Chromatographic separation and mass spectrometric detection were performed under optimized

analytical conditions. Acquired data (MassLynx V4.2) were processed through Progenesis QI software for feature extraction, peak alignment, and data normalization. Metabolite identification was achieved by cross-referencing with the METLIN database, public repositories, and a custom-established Baimaike database, complemented by theoretical fragmentation pattern analysis. Mass accuracy thresholds were maintained at 100 ppm for precursor ions and 50 ppm for product ions. The PCA analysis and orthogonal partial least squares discriminant analysis (OPLS-DA) were conducted using the BMKCloud (https://international.biocloud.net/zh/dashboard). Differential metabolite screening was performed based on multivariate and univariate statistical analyses. The Variable Importance in Projection (VIP) values derived from the OPLS-DA model were initially employed to identify potential metabolic differences between cultivars or tissues. Subsequently, these findings were integrated with univariate analysis results (fold change $\geqslant$ 1 and P-value $<$ 0.05) to enhance the reliability of differential metabolite selection. The screening criteria were set at VIP $\geqslant$ 1, FC $\geqslant$ 1, and P $<$ 0.05. The final results were visualized using a volcano plot. The cluster profile package was employed to perform an enrichment analysis of KEGG annotation results using the hypergeometric test, and an enrichment dot plot was created.

2.7 Integrative Investigation of Transcriptional and Metabolic Networks

Comprehensive bioinformatics analysis of gene expression and metabolic profiles was carried out utilizing the BMK Cloud analytical suite (www.biocloud.net), including library quality assessment, principal component analysis, differential

expression analysis, gene function and metabolic pathway annotation and functional enrichment, and combined analysis.

3. Results

3.1 Comparison of Melanin Content in Breast Muscle

Figure 1(A) was a comparison of high and low levels of melanogenesis and subsequent deposition in the breast muscle of Muchuan Black Bones Chickens. As illustrated in Figure 1(B), black bones chickens were selected for the ML group (low melanin expression group) and the MH group (high melanin expression group) based on their melanin content, the expression levels of melanin in breast muscle were (103.193 ± 3.598) mg/mL and (121.731 ± 3.69) mg/mL, respectively. Statistical analysis revealed a significantly elevated melanin concentration in the pectoral muscle tissue of the high-level group compared with the low-level group ($P < 0.01$).

3.2 Transcriptome Analysis

3.2.1 Raw Sequencing Data Quality Assessment Summary

Table 1 presents a summary of the RNA-Seq results from 10 Muchuan Black Bones chicken samples, comprising five ML and five MH samples, along with both raw and clean reads. The Q30 value exceeded 90.43%, while the GC content ranged from 52.36% to 53.38%.

3.2.2 Gene Differential Expression Analysis

Transcriptomic analysis revealed significant differential gene expression patterns between high and low melanin groups, identifying 73 DEGs (54 upregulated and 19 downregulated), as illustrated in Figure 1(C). The top ten DEGs based on |log2(FC)|

were presented in Table 2.

3.2.3 Data Quality Assessment of qPCR

Figure 1(D) illustrates the expression trends of the two differentially expressed genes as determined by transcriptome sequencing and real-time quantitative PCR. The figure shows that the RNA-seq expression level of the *GIPC2* gene in the MH group was approximately 5-fold higher than that in the ML group, while the qPCR expression level in the MH group was 4.5-fold higher. Similarly, for the *CREB5* gene, the RNA-seq expression level in the MH group was 5-fold higher compared to the ML group, and the qPCR expression level was 3.1-fold higher. These results demonstrate a largely consistent expression pattern between the two methods.

3.2.4 GO Enrichment Analysis of DEGs

The classification of differentially expressed genes (DEGs) from the high and low melanin content groups into GO terms revealed significant enrichment ($P < 0.05$) across 6 functional categories. Biological processes (BP) included auditory receptor cell stereocilium organization and axon development. Cellular components (CC) encompassed dendrites, integral plasma membrane components, and plasma membranes. Molecular functions (MF) were characterized by integrin binding, as illustrated in Figure 1(E).

3.2.5 KEGG Enrichment Analysis of DEGs

The KEGG pathway enrichment analysis identified two metabolic pathways with significant enrichment ($P < 0.05$), including the neuroactive ligand-receptor interaction pathway and the arginine-proline metabolism pathway, as demonstrated in

Figure 1(F). Pathways associated with pigmentation include adrenergic signaling in cardiomyocytes, the calcium signaling pathway, and the mitogen-activated protein kinase (MAPK) signaling pathway. Specifically, the adrenergic signaling pathway in cardiomyocytes involves the tropomyosin 2 (*TPM2*) gene and cAMP-responsive element binding protein 5 (*CREB5*) gene; the calcium signaling pathway includes histamine receptor H2 (*HRH2*) gene and fibroblast growth factor 1 (*FGF1*) gene; while the MAPK signaling pathway encompasses dual specificity phosphatase 1 (*DUSP1*) gene and fibroblast growth factor 1 (*FGF1*) gene.

3.3 Metabolome Analysis

3.3.1 PCA Analysis

Figure 2 illustrates the distribution of samples along PC1 and PC2, from which it was inferred that the MH and ML groups exhibited significant differences in metabolic composition.

3.3.2 OPLS-DA Analysis

To enhance the identification of differential metabolites, this study also performed orthogonal partial least squares discriminant analysis (OPLS-DA). As illustrated in Figure 3(A), samples from the MH and ML groups were distinctly separable along the t1 axis. To mitigate overfitting, the model was verified in this experiment, as shown in Figure 3(B). The figure demonstrates that R2Y values predominantly cluster around 1, indicating that the model was robust and not subject to overfitting.

3.3.3 Differential Metabolite Analysis

Figure 4(A) displays the metabolomic screening results, identifying 234 differentially abundant metabolites, among which 158 were significantly downregulated, while 76 were upregulated. Among the downregulated metabolites highlighted, several notable compounds include Glucoiberverin, CDP-ethanolamine, m-Trigallic acid, Uridine diphosphate glucuronic acid, and UDP-4-dehydro-6-deoxy-D-glucose.

3.3.3 KEGG Enrichment Analysis of Differential Metabolites

Figure 4(B) showed that pathways such as histidine metabolism and terpenoid backbone biosynthesis were involved. Histidine metabolism, Terpenoid backbone biosythesis and Glycerophospholipid metabolism were significantly enriched in the metabolic pathway.

3.4 Integrated Transcriptome and Metabolome Analysis

3.4.1 Weighted Gene Co-expression Network Analysis (WGCNA)

This study conducted WGCNA analysis on the transcriptome and metabolome data independently, revealing that certain modules, such as MEdarkmagenta and GEmagenta, exhibited strong positive correlations in the heatmap (Figure 5(A)). Conversely, a strong negative correlation was observed between MElightyellow and GEdarkgreen.

3.4.2 Bidirectional Orthogonal Partial Least Squares Analysis

Joint Score 1 accounts for the maximum shared variation between transcriptomic and metabolomic data, whereas Joint Score 2 captures the secondary shared variation. Figure 6(A) illustrates that the distribution of W8 (red) was relatively close to that of

B8 (blue).

Based on the results of element loading values, this study identified the top 15 differential genes and metabolites with the highest loading values in the first two principal components and constructed bar charts (Figure 6(B)) to illustrate those with the strongest associations. Certain genes exhibit particularly high negative loading values on Joint Loading 1. specific compounds, such as Semivioxanthin and Gossypol, demonstrate significant positive or negative loading values within the model, highlighting their crucial roles in metabolomic data.

4. Discussion

The deposition of melanin in Muchuan Black Bones chickens significantly influences the appearance quality, with higher melanin deposition levels correlating to increased nutritional value[26]. Although some progress has been made in breeding studies of Muchuan Black Bones Chickens in recent years, the existing studies mainly focused on the selection of black skin traits, focused on appearance and market value, while the selection of melanin deposits in the breast muscle was relatively rare, and the existing data are mostly studies of older black bones chickens[27]. Research in this field has not fully explored the specific effects of melanin deposition in the breast muscle on appearance quality characteristics, especially the regulatory mechanism of melanin deposition at different growth stages and its potential in appearance quality enhancement. Through the previous observation, it was found that the melanin deposition in the early growth stage (35 days of age) had shown a significant degree of separation, especially in the breast muscle and skin[28]. Therefore, the early stage

of melanin deposition needs to be further explored, especially through breeding strategies to optimize its impact on the appearance quality.

The transcriptomic analysis revealed that two genes associated with melanin synthesis, namely *GIPC2* and *CREB5*, were significantly up-regulated. Previous studies have demonstrated that the missense mutations D125N and E288K in *GIPC2* were implicated in malignant melanoma development[29]. As a transcription factor belonging to the *CREB* family, *CREB5* exerts its regulatory function by specifically interacting with cAMP response elements (CREs) in downstream genes[30], thereby playing a regulatory role in various cell-specific biological activities, including cell growth, survival, metabolism, and differentiation. It has been established that members of the *CREB* family can enhance melanogenesis through microphthalmia-associated transcription factor (*MITF*) activation; however, the specific role of *CREB5* in this context remains inadequately characterized. As an integral component of the *CREB* family, *CREB5* may indirectly influence *MITF* expression via analogous mechanisms[11]. A critical regulatory pathway for melanogenesis was the cAMP response elements (cAMP-PKA) signaling cascade, which can be further modulated by activating *CREB* family transcription factors. Consequently, *CREB5* may play a significant role in this pathway and impact the expression of downstream genes associated with melanogenesis.

In the differential metabolite analysis, this study identified Glucoiberverin and Uridine diphosphate glucuronic acid (UDP glucuronic acid) as key metabolites that are significantly associated with melanin deposition. These metabolites were

primarily linked to cellular metabolism, which plays a crucial role in regulating various biochemical processes that can influence melanin accumulation. Glucoiberverin is a natural sulfur-containing glucosinolate which can be enzymatically hydrolyzed into sulforaphane, a compound known to reduce cell viability and modulate the expression levels of various histone deacetylases. Through this mechanism, sulforaphane regulates the acetylation and methylation of specific lysine residues on histones H3 and H4 in malignant melanoma cells[31]. While neither Glucoiberverin nor UDP-glucuronic acid plays a crucial role in key melanogenic processes, particularly in facilitating the bioconversion of tyrosine to melanin pigments, their contributions to cellular energy metabolism and metabolic regulation were vital for the overall process of melanin deposition. UDP-glucuronic acid is a key player in glycosylation reactions, particularly in the biosynthesis of glycosaminoglycans and the detoxification processes within the cell[32]. This metabolite helps in the conjugation of various metabolites to improve their solubility and excretion, indirectly influencing cellular function. Its role in glycosylation also extends to the modification of proteins and lipids that may be involved in the regulation of melanin-producing enzymes or the structural integrity of melanocytes. The ability of UDP-glucuronic acid to influence the glycosylation of melanogenic enzymes or cellular receptors could thereby affect the efficiency and regulation of melanin synthesis, influencing the overall deposition of melanin in tissues[33]. Although these metabolites do not directly participate in the melanogenic biochemical pathway, they play pivotal roles in modulating cellular homeostasis, maintaining

energy metabolism, and regulating protein post-translational modifications, thereby exerting substantial influence on melanin deposition processes. This finding underscores the crucial role of metabolic intermediates in regulating pigment biosynthesis, providing novel research perspectives for investigating the intricate relationship between metabolic networks and phenotypic pigmentation characteristics, particularly in economically important poultry species such as the Muchuan Black Bones chickens. These indirect roles may also offer insights into improving the nutritional value and quality of appearance based on melanin content. In addition, differential metabolomic analysis revealed significant enrichment of metabolites such as CDP-ethanolamine, m-trigallic acid, and UDP-4-dehydro-6-deoxy-D-glucose. CDP-ethanolamine (cytidine diphosphate ethanolamine) is a key intermediate in the Kennedy pathway, which is primarily involved in the biosynthesis of phosphatidylcholine (PE)[34]. PE is a major phospholipid component of cellular and organelle membranes, playing crucial roles in maintaining membrane fluidity, membrane protein function, and intracellular signal transduction, all of which are important for cellular homeostasis in livestock tissues. Information on m-trigallic acid is currently limited, and its biological role remains unclear, necessitating further investigation to determine its potential physiological significance in animal systems. UDP-4-dehydro-6-deoxy-D-glucose is a sugar-nucleotide intermediate involved in the biosynthesis of deoxy sugars in microorganisms and plants. Although its function in animals is not well characterized, its enrichment in this study suggests potential metabolic relevance. However, their significant differential abundance in this study

suggests they may participate in melanin-related metabolic regulation, warranting further mechanistic investigation.

According to the metabolomics, the KEGG was analysed for two pathways: phenylalanine, tyrosine, and tryptophan biosynthesis. Phenylalanine is enzymatically converted to tyrosine by phenylalanine hydroxylase (PAH), after which tyrosine undergoes further oxidation to form dihydroxyphenylalanine (DOPA) through the action of tyrosinase[35]. Subsequently, DOPA is transformed into dopachrome via a series of intricate chemical reactions, ultimately yielding eumelanin or pheomelanin, which represent the primary types of melanin[36]. A substantial number of studies have identified key genes involved in melanin biosynthesis and pigmentation regulation, often referred to as "marker genes" in melanogenesis. These included *EDNRB2, EDN3, TYR, TYRP1, MITF,* and *PMEL17*, which played crucial roles in different stages of melanogenesis, ranging from melanoblast migration to enzymatic catalysis and melanosome formation. However, these genes were not detected in this study due to sequencing limitations, thus, low-expressed genes might not have been effectively captured. Additionally, external factors such as diet, light exposure, and temperature could have influenced melanin production, leading to changes in gene expression patterns.

5. Conclusion

This study identified *GIPC2* and *CREB5* genes and CDP-ethanolamine, m-trigallic acid, UDP-4-dehydro-6-deoxy-D-glucose, and UDP glucuronic acid related to melanin deposition, the phenylalanine, tyrosine, and tryptophan

biosynthesis pathways, which influence melanin formation.

Disclosures

The authors declare that they have no competing interests.

Funding

This research was funded by the Guangdong Basic and Applied Basic Research Foundation (2022A1515111078) and Guizhou Science and Technology Key Project ([2022] No. 034).

Author Contributions

Conceptualization, experimental design: ZHANG Zheng-fen, YE Fei, LI Hua, MA Zheng, XIANG Hai. Manuscript writing:YE Fei, LIU Wei, and QI Hao . Conducting experiments: LIU Wei, YAO Xiu-mei, YIN Qiong, JIE Hong-wei. Data analysis: LIU Wei and LI Liang. All authors have read and approved the final version of the manuscript.

Acknowledgments

We would like to express sincere thanks to the Analysis and Testing Center of Foshan University for their great support with the testing equipment.

Availability of Data and Materials

The data that support the findings of this study have been deposited into the CNGB Sequence Archive (CNSA) of China National GeneBank DataBase (CNGBdb) with accession number CNP0006904.

References

[1] L. C. Lin and W. T. Chen, "The study of antioxidant effects in melanins extracted from various tissues of animals," *Asian Australas. J. Anim. Sci.*, vol. 18, no. 2, pp. 277–

281, Feb. 2005, doi: 10/gqt44n.

[2] W. Q. Zhu *et al.*, “Molecular genetic diversity and maternal origin of Chinese black-bone chicken breeds,” *Genet Mol Res*, vol. 13, no. 2, pp. 3275–3282, Apr. 2014, doi: 10/f58dvh.

[3] S.-R. Chen, B. Jiang, J.-X. Zheng, G.-Y. Xu, J.-Y. Li, and N. Yang, “Isolation and characterization of natural melanin derived from silky fowl (*Gallus gallus domesticus Brisson*),” *Food Chem.*, vol. 111, no. 3, pp. 745–749, Dec. 2008, doi: 10/ftt548.

[4] Y. Tian, M. Xie, W. Wang, H. Wu, Z. Fu, and L. Lin, “Determination of carnosine in Black-Bone Silky Fowl (*Gallus gallus domesticus* Brisson) and common chicken by HPLC,” *Eur. Food Res. Technol.*, vol. 226, no. 1–2, pp. 311–314, Nov. 2007, doi: 10/fcrv4n.

[5] L. Guo *et al.*, “Recent advances and progress on melanin: From source to application,” *Int J Mol Sci*, vol. 24, no. 5, p. 4360, Feb. 2023, doi: 10.3390/ijms24054360.

[6] S. J. Orlow, M. P. Osber, and J. M. Pawelek, “Synthesis and characterization of melanins from dihydroxyindole-2-carboxylic acid and dihydroxyindole,” *Pigment Cell Res*, vol. 5, no. 3, pp. 113–121, Sep. 1992, doi: 10.1111/j.1600-0749.1992.tb00007.x.

[7] L. Li *et al.*, “Endothelin receptor B2 (EDNRB2) gene is associated with spot plumage pattern in domestic ducks (anas platyrhynchos),” *PLoS One*, vol. 10, no. 5, p. e0125883, 2015, doi: 10.1371/journal.pone.0125883.

[8] A. Shinomiya *et al.*, “Gene duplication of endothelin 3 is closely correlated with the hyperpigmentation of the internal organs (fibromelanosis) in silky chickens,” *Genetics*, vol. 190, no. 2, pp. 627–638, Feb. 2012, doi: 10.1534/genetics.111.136705.

[9] D. Li *et al.*, “Transcriptome Analysis of the Breast Muscle of Xichuan Black-Bone Chickens Under Tyrosine Supplementation Revealed the Mechanism of Tyrosine-Induced Melanin Deposition,” *Front Genet*, vol. 10, p. 457, 2019, doi: 10/gqvzbz.

[10] D. Fang, Y. Tsuji, and V. Setaluri, “Selective down-regulation of tyrosinase family gene TYRP1 by inhibition of the activity of melanocyte transcription factor, MITF,” *Nucleic Acids Res*, vol. 30, no. 14, pp. 3096–3106, Jul. 2002, doi: 10.1093/nar/gkf424.

[11] W. J. Yun *et al.*, “Microtubule-associated protein light chain 3 is involved in melanogenesis via regulation of MITF expression in melanocytes,” *Sci. Rep.*, vol. 6, p. 19914, Jan. 2016, doi: 10/f77tg5.

[12] L. Keeling *et al.*, “Chicken genomics: Feather-pecking and victim pigmentation,” *Nature*, vol. 431, no. 7009, pp. 645–646, Oct. 2004, doi: 10.1038/431645a.

[13] C. Sarkar *et al.*, “Human placental protein/peptides stimulate melanin synthesis by enhancing tyrosinase gene expression,” *Mol Cell Biochem*, vol. 285, no. 1–2, pp. 133–142, Apr. 2006, doi: 10.1007/s11010-005-9069-3.

[14] S. Yu, G. Wang, and J. Liao, “Association of a novel SNP in the ASIP gene with skin color in black-bone chicken,” *Anim Genet*, vol. 50, no. 3, pp. 283–286, Jun. 2019, doi: 10/gtxfgn.

[15] J. Bai *et al.*, “Integrated transcriptomics and proteomics assay identifies the role of FCGR1A in maintaining sperm fertilization capacity during semen cryopreservation in sheep,” *Front. Cell Dev. Biol.*, vol. 11, p. 1177774, 2023, doi: 10/gt68gc.

[16] M. Szczepanek, A. Frątczak, and A. Lis-Święty, "Narrow-band reflectance spectrophotometry for the assessment of erythematous and hyperpigmented skin lesions in localized scleroderma: A preliminary study," *Clin Cosmet Investig Dermatol*, vol. 14, pp. 575–580, 2021, doi: 10.2147/CCID.S312208.
[17] S. Ito, "High-performance liquid chromatography (HPLC) analysis of eu- and pheomelanin in melanogenesis control," *J Invest Dermatol*, vol. 100, no. 2 Suppl, pp. 166S-171S, Feb. 1993.
[18] S. Kalia, J. Zhao, H. Zeng, D. McLean, N. Kollias, and H. Lui, "Melanin quantification by in vitro and in vivo analysis of near-infrared fluorescence," *Pigment Cell Melanoma Res*, vol. 31, no. 1, pp. 31–38, Jan. 2018, doi: 10.1111/pcmr.12624.
[19] L. Zhang *et al.*, "Biotransformation of ginsenoside Rb1 and rd to four rare ginsenosides and evaluation of their anti-melanogenic effects," *J Nat Med*, vol. 77, no. 4, pp. 939–952, Sep. 2023, doi: 10.1007/s11418-023-01719-5.
[20] M. Ferguson-Pell and S. Hagisawa, "An empirical technique to compensate for melanin when monitoring skin microcirculation using reflectance spectrophotometry," *Med Eng Phys*, vol. 17, no. 2, pp. 104–110, Mar. 1995, doi: 10.1016/1350-4533(95)91880-p.
[21] Y. Deng, X. Qu, Y. Yao, M. Li, C. He, and S. Guo, "Investigating the impact of pigmentation variation of breast muscle on growth traits, melanin deposition, and gene expression in Xuefeng black-bone chickens," *Poult Sci*, vol. 103, no. 6, p. 103691, Jun. 2024, doi: 10/gtxf75.
[22] Z. Yao, L. Guo, L. Zhang, and Q. Nie, "Transcriptome and Metabolome Profiling Provide New Insights into Disuse Muscle Atrophy in Chicken: The Potential Role of Fast-Twitch Muscle Fibers," *Int. J. Mol. Sci.*, 2024.
[23] A. S. Thind *et al.*, "Demystifying emerging bulk RNA-Seq applications: the application and utility of bioinformatic methodology," *Brief. Bioinform.*, vol. 22, no. 6, p. bbab259, Nov. 2021, doi: 10/gmdhhz.
[24] A. Conesa *et al.*, "A survey of best practices for RNA-seq data analysis," *Genome Biol.*, vol. 17, p. 13, Jan. 2016, doi: 10/f3vhpj.
[25] O. R. Alara, N. H. Abdurahman, and C. I. Ukaegbu, "Extraction of phenolic compounds: A review," *Curr. Res. Food Sci.*, vol. 4, pp. 200–214, 2021, doi: 10/grr42c.
[26] F. Ma, L. Luo, and X. Gao, "Metabolite and transcriptome analyses revealed the modulation of fructo-oligosaccharide on ileum metabolism of Taiping chickens," *J Appl Microbiol*, vol. 132, no. 3, pp. 2249–2261, Mar. 2022, doi: 10/gtxfcd.
[27] H. Wang *et al.*, "Genetics and breeding of a black-bone and blue eggshell chicken line. 1. Body weight, skin color, and their combined selection," *Poult. Sci.*, vol. 100, no. 5, p. 101035, May 2021, doi: 10/gt68g6.
[28] J. Liu, T. Zhang, L. Fu, H. Li, L.-Q. Huang, and Y. Yuan, "[Review of genetic diversity and breed identification of black-bone silky fowl]," *Zhongguo Zhong Yao Za Zhi = Zhongguo Zhongyao Zazhi = China Journal of Chinese Materia Medica*, vol. 47, no. 8, pp. 2021–2027, Apr. 2022, doi: 10/gqpf52.
[29] M. Katoh, "Functional proteomics, human genetics and cancer biology of GIPC family members," *Exp Mol Med*, vol. 45, no. 6, p. e26, Jun. 2013, doi: 10/gmjmck.
[30] C. Bian, R. Li, Z. Wen, W. Ge, and Q. Shi, "Phylogenetic Analysis of Core Melanin

Synthesis Genes Provides Novel Insights Into the Molecular Basis of Albinism in Fish," *Front. Genet.*, vol. 12, Aug. 2021, doi: 10/g7mqgr.
[31] M. Mitsiogianni, D. T. Trafalis, R. Franco, V. Zoumpourlis, A. Pappa, and M. I. Panayiotidis, "Sulforaphane and iberin are potent epigenetic modulators of histone acetylation and methylation in malignant melanoma," *Eur J Nutr*, vol. 60, no. 1, pp. 147–158, Feb. 2021, doi: 10.1007/s00394-020-02227-y.
[32] J. J. Hjelle, "Hepatic UDP-glucuronic acid regulation during acetaminophen biotransformation in rats," *J. Pharmacol. Exp. Ther.*, vol. 237, no. 3, pp. 750–756, Jun. 1986.
[33] R. W. Dellinger and F. L. Meyskens, "Detection of total UDP-glucuronosyltransferase (UGT) activity in melanoma cells," Totowa, NJ: Humana Press, pp. 1–9. doi: 10.1007/7651_2015_298.
[34] F. Gibellini and T. K. Smith, "The kennedy pathway--de novo synthesis of phosphatidylethanolamine and phosphatidylcholine," *IUBMB Life*, vol. 62, no. 6, pp. 414–428, Jun. 2010, doi: 10.1002/iub.337.
[35] Z. Rzepka, E. Buszman, A. Beberok, and D. Wrześniok, "From tyrosine to melanin: Signaling pathways and factors regulating melanogenesis," *Postepy Higieny I Medycyny Doswiadczalnej (Online)*, vol. 70, no. 0, pp. 695–708, Jun. 2016, doi: 10/gjgqct.
[36] L. Luo *et al.*, "The amino acid transporter SLC16A10 promotes melanogenesis by facilitating the transportation of phenylalanine," *Exp. Dermatol.*, vol. 33, no. 8, p. e15165, Aug. 2024, doi: 10/g7rs9t.

Figure 1. (A) Comparison of blackness between MH and ML black bones chickens.

Click here to access/download;Figure;Fig1.(A).tif

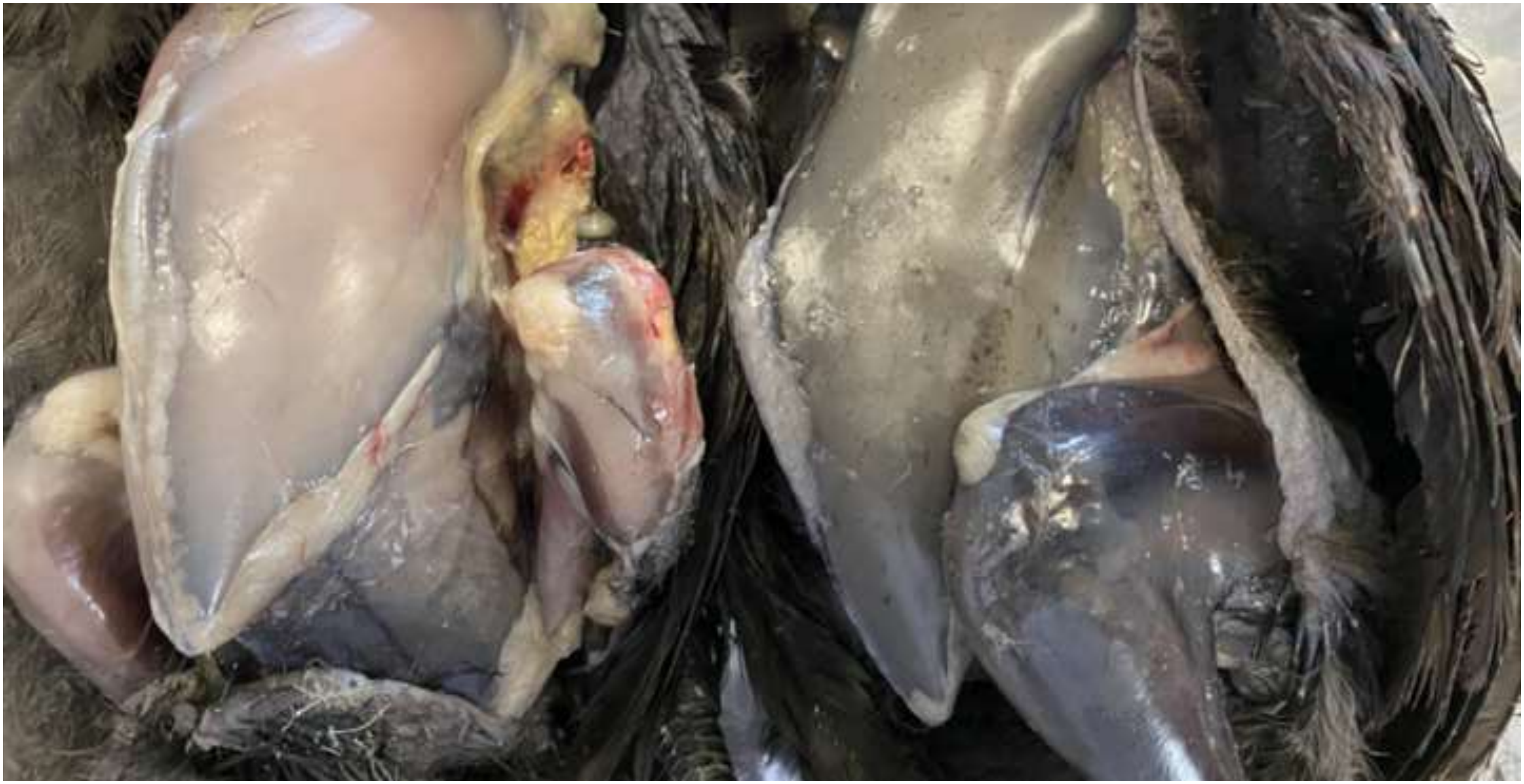

Figure 1. (B) Statistical significance was denoted as follows: *P < 0.05 (significant) and **P < 0.01 (highly significant).

Click here to access/download;Figure;Fig1.(B).tif

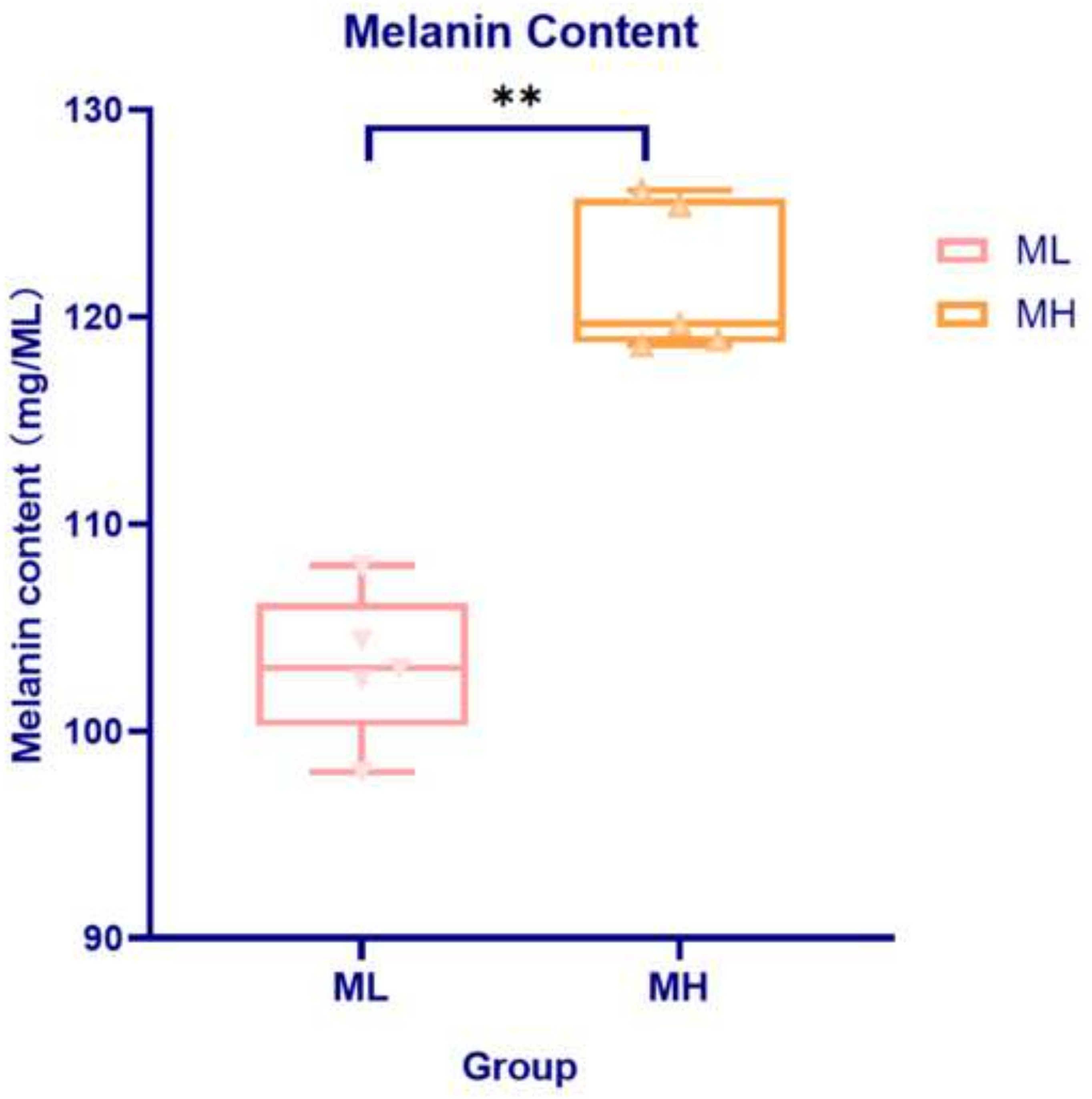

Figure 1. (C) DEGs volcano map.

Click here to access/download;Figure;Fig1.(C).tif

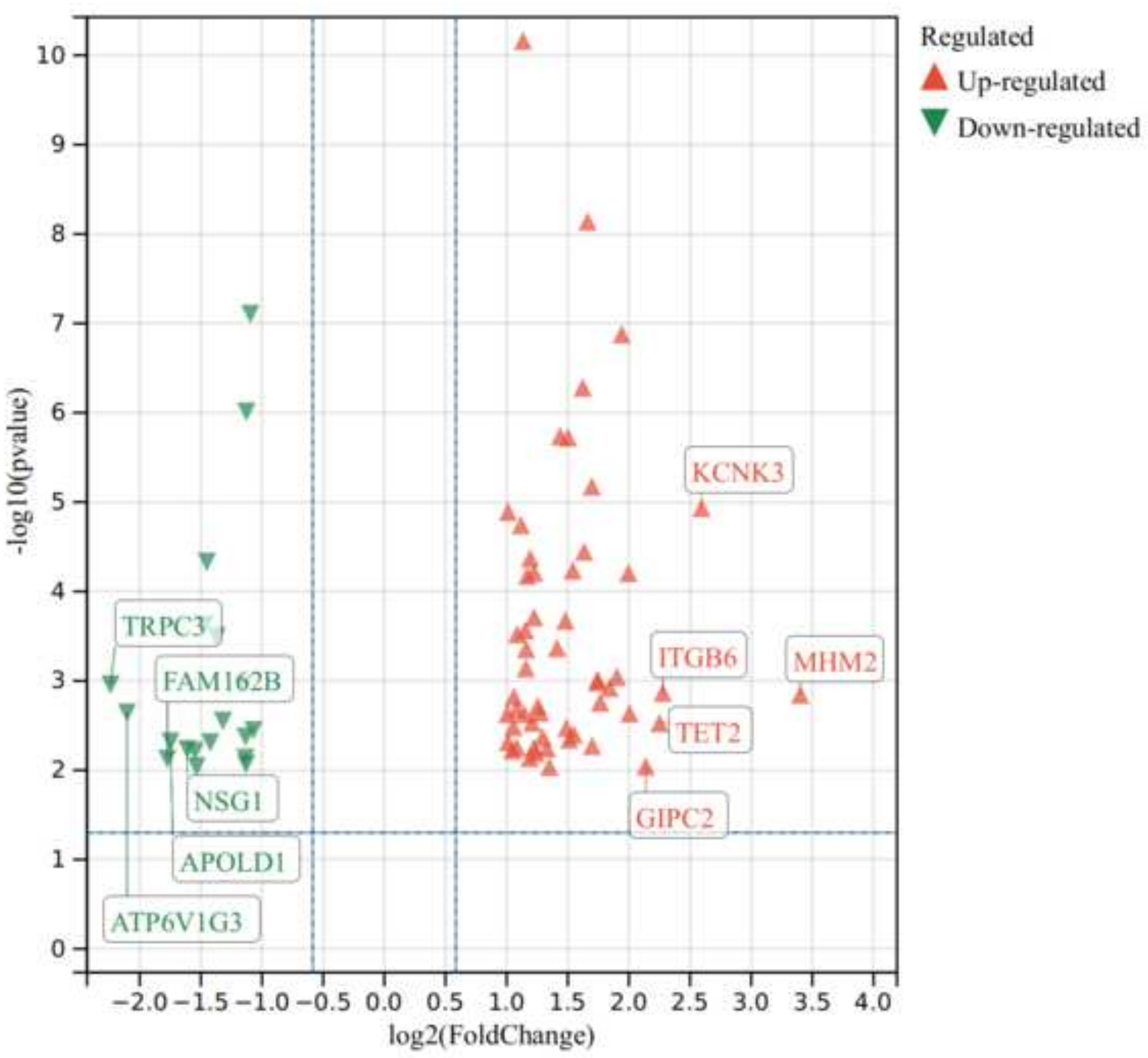

Figure 1. (D) Expression trend of differential gene transcriptome sequencing and real-time fluorescence quantitative PCR.

Click here to access/download;Figure;Fig1.(D).tif

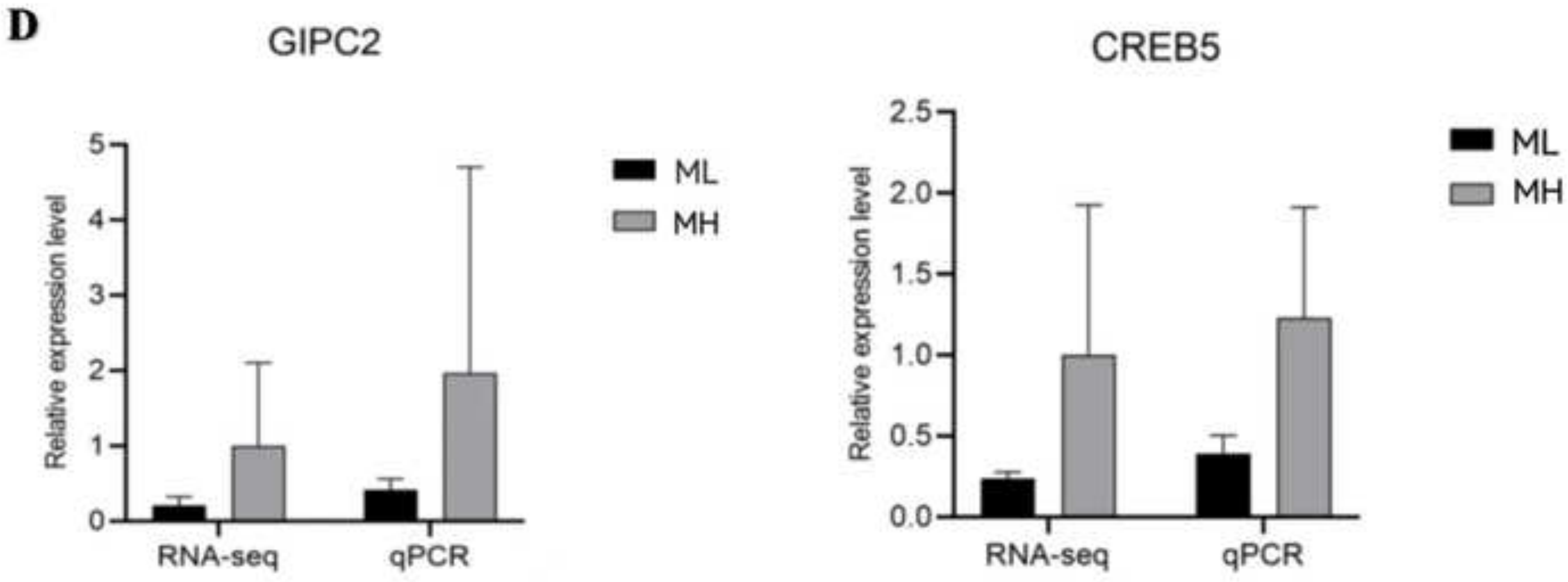

Figure 1. (E) Functional enrichment of DEGs GO.

Click here to access/download;Figure;Fig1.(E).tif

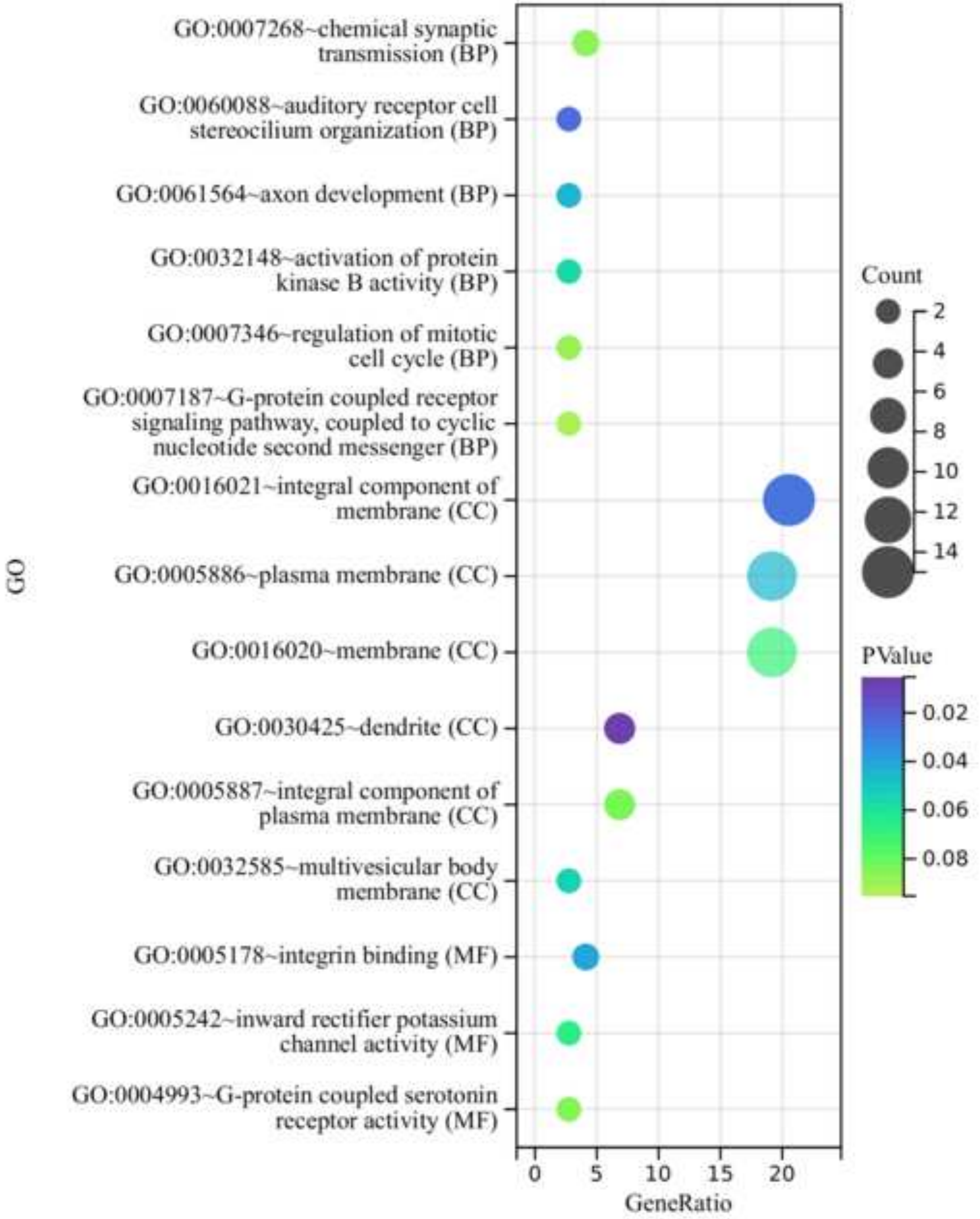

Figure 1.(F)Enrichment of DEGs KEGG pathway.

Click here to access/download;Figure;Fig1.(F).tif

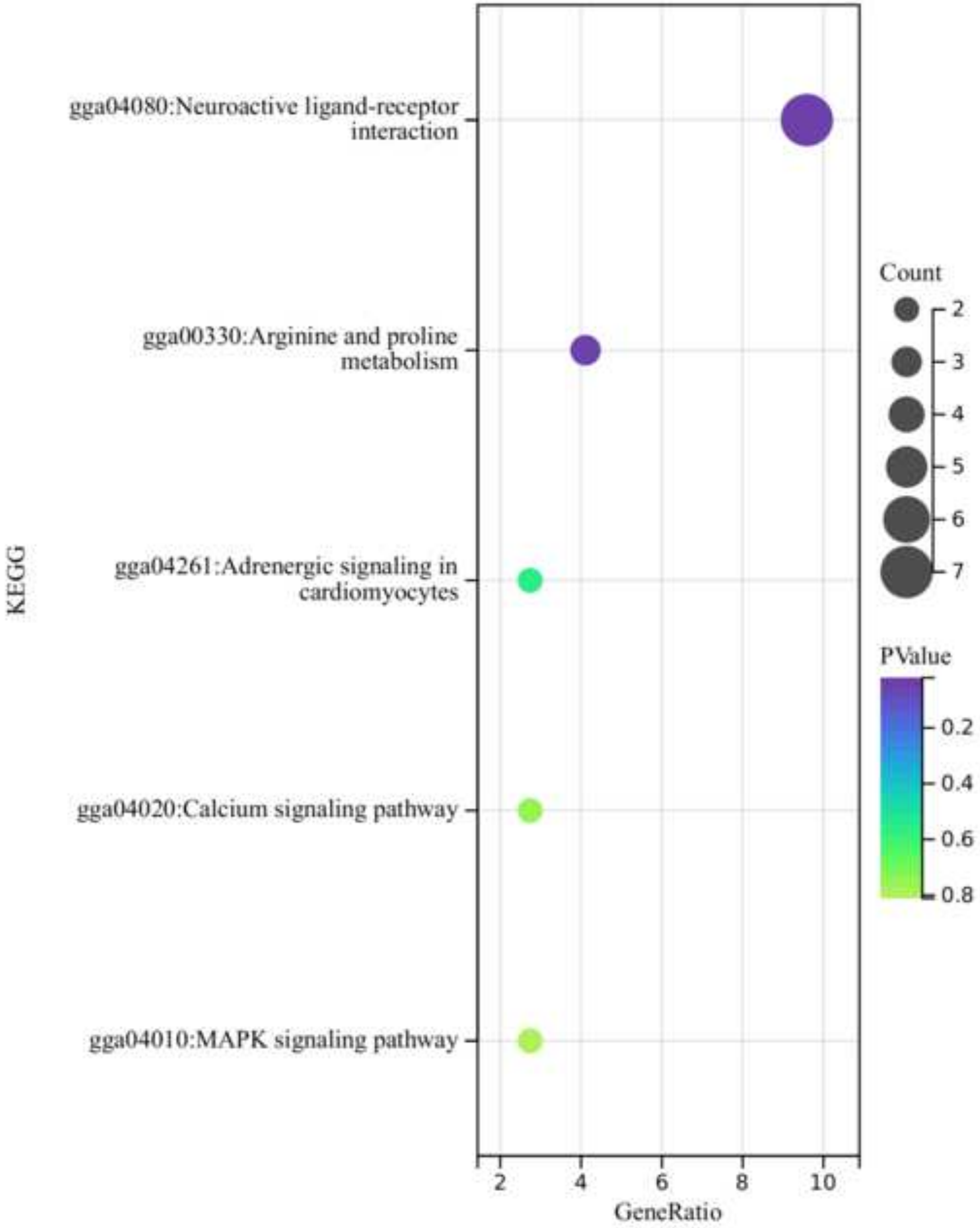

Figure 2. PCA analysis of all samples in two dimensions.

Click here to access/download;Figure;Fig2.tif

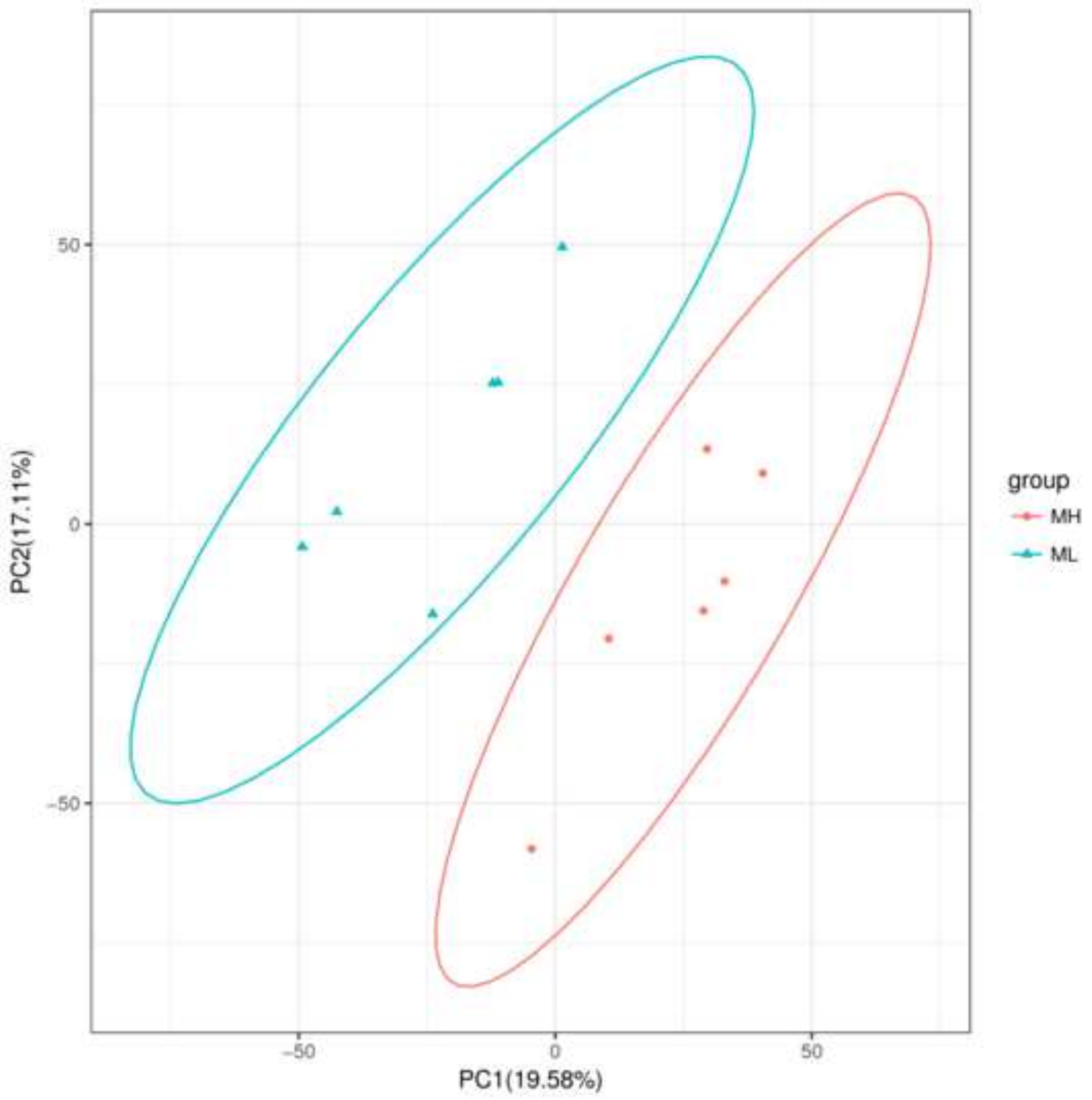

Figure 3. (A) Orthogonal Partial Least Squares Discriminant Analysis (OPLS-DA).

Click here to access/download;Figure;Fig3.(A).tif

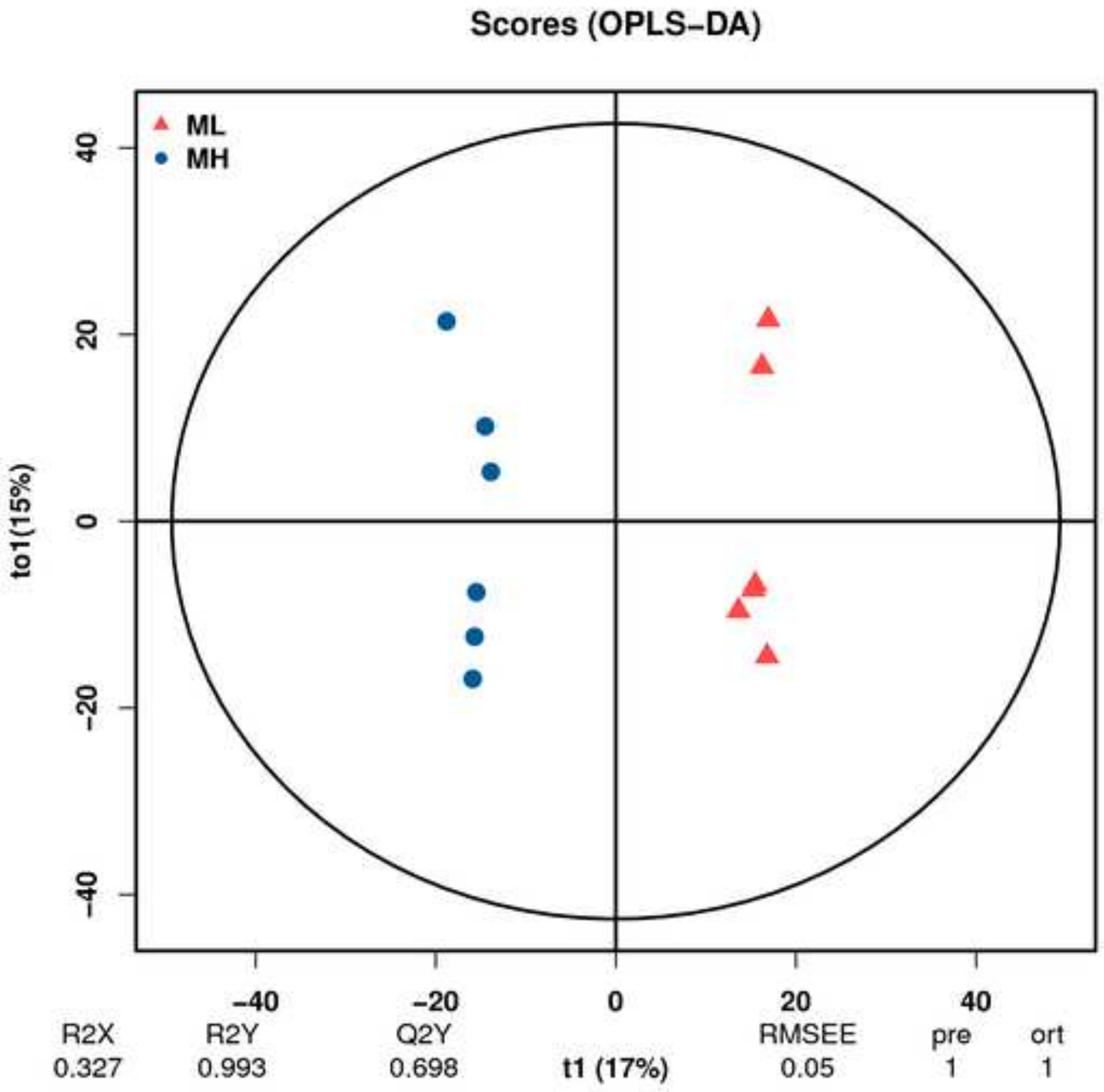

Figure3. (B) OPLS-DA model replacement test diagram

Click here to access/download;Figure;Fig3.(B).tif

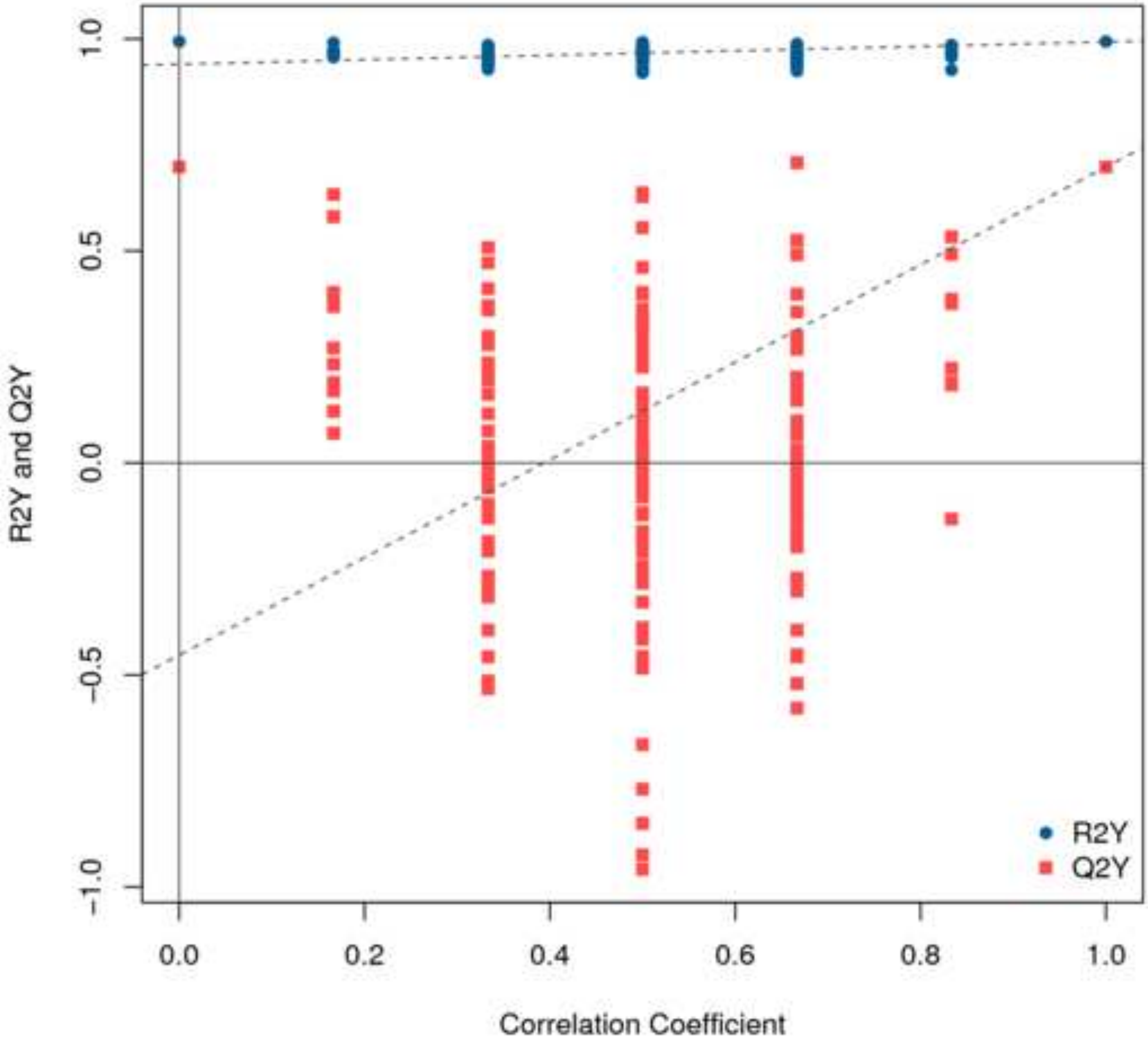

Figure 4. (A) Volcanic map of differential metabolites.

Click here to access/download;Figure;Fig4.(A).tif

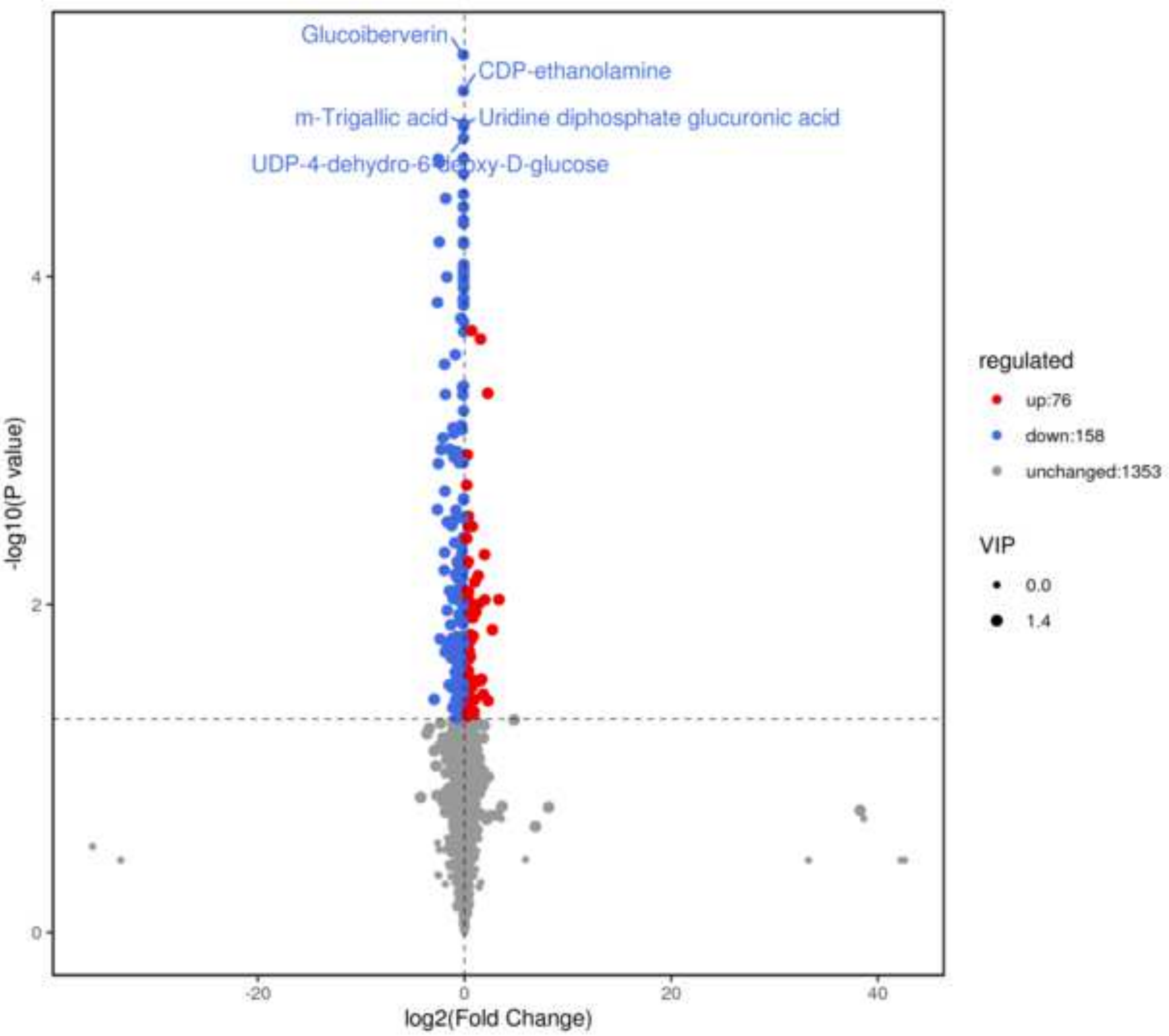

Figure 4. (B) KEGG enrichment map of differential metabolites.

Click here to access/download;Figure;Fig4.(B).tif

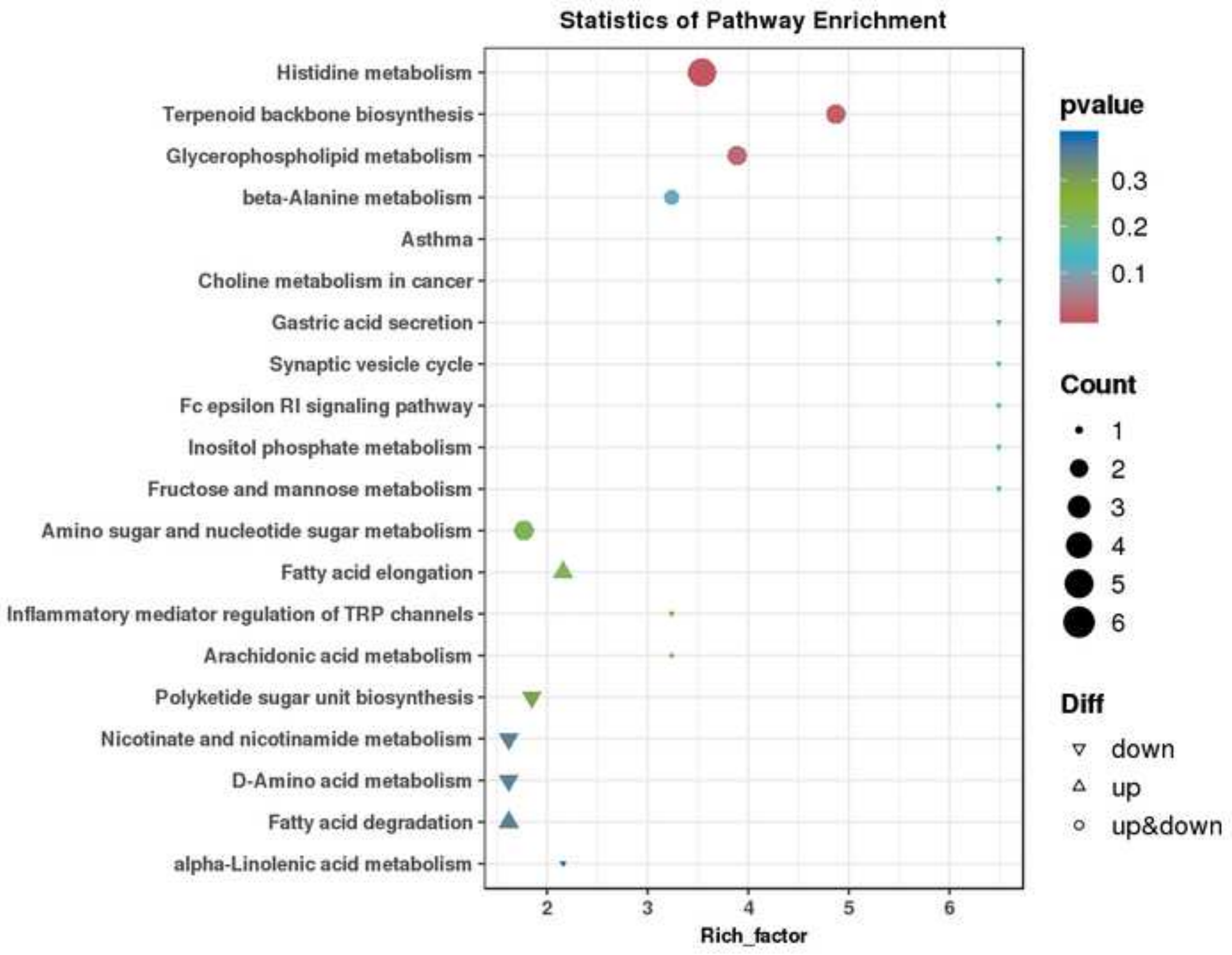

Figure 5. (A)Weighted gene Co-expression network analysis (WGCNA). Click here to access/download;Figure;Fig5.(A).tif

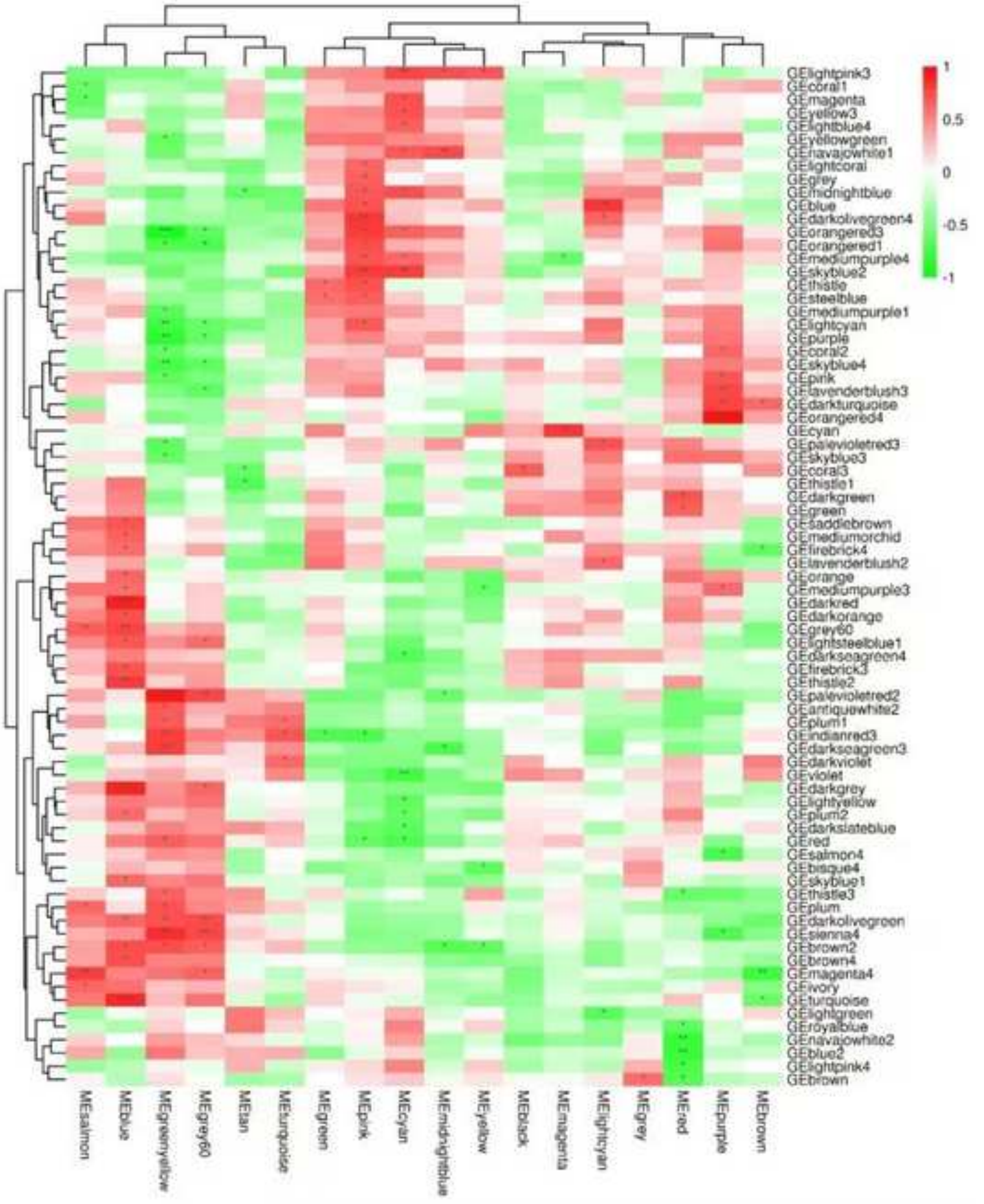

Figure 6. (A)ML vs MH O2PLS scores.

Click here to access/download;Figure;Fig6.(A).tif

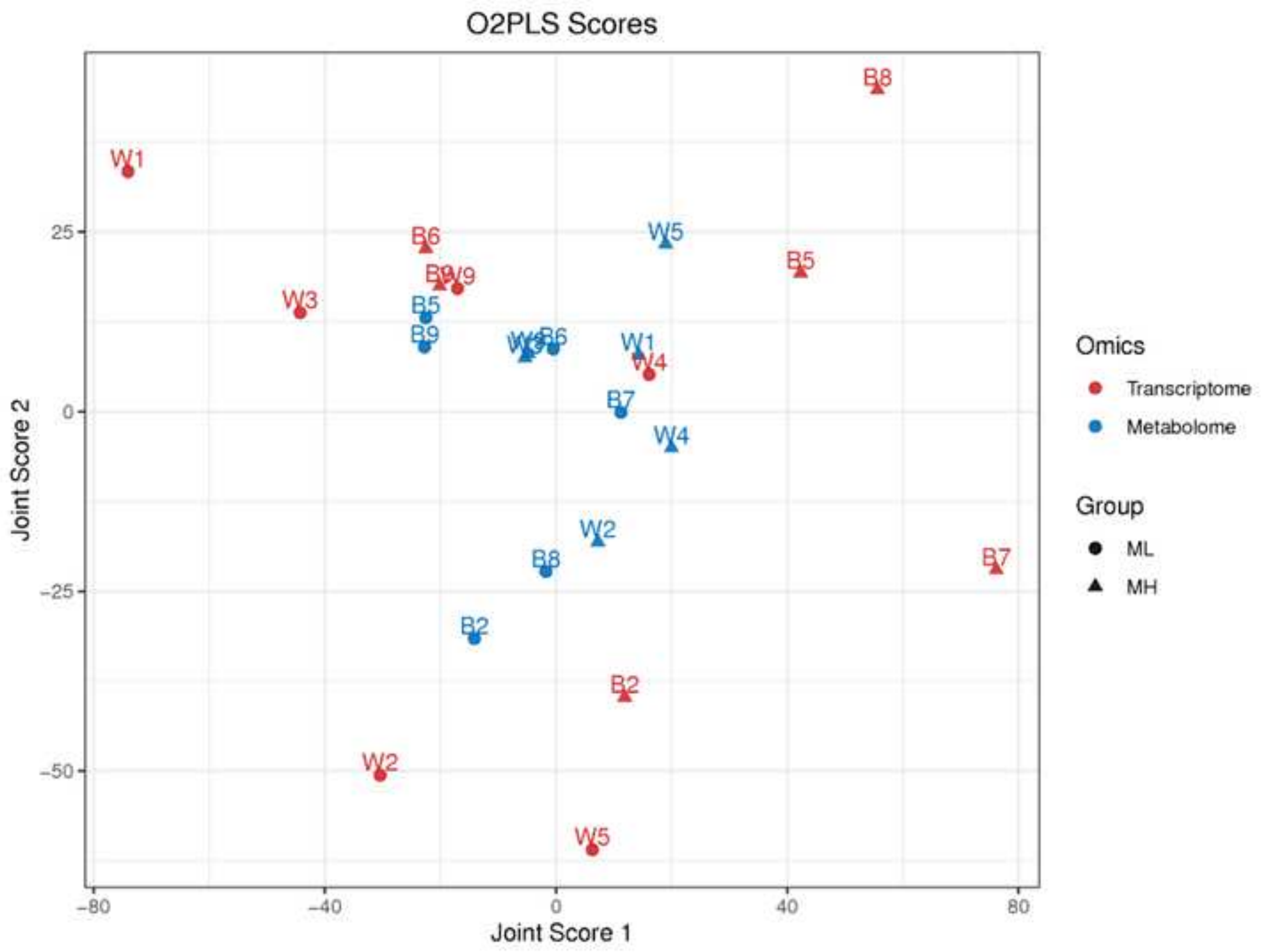

Figure 6.(B)ML vs MH top15 O2PLS loadings bar.

Click here to access/download;Figure;Fig6.(B).tif

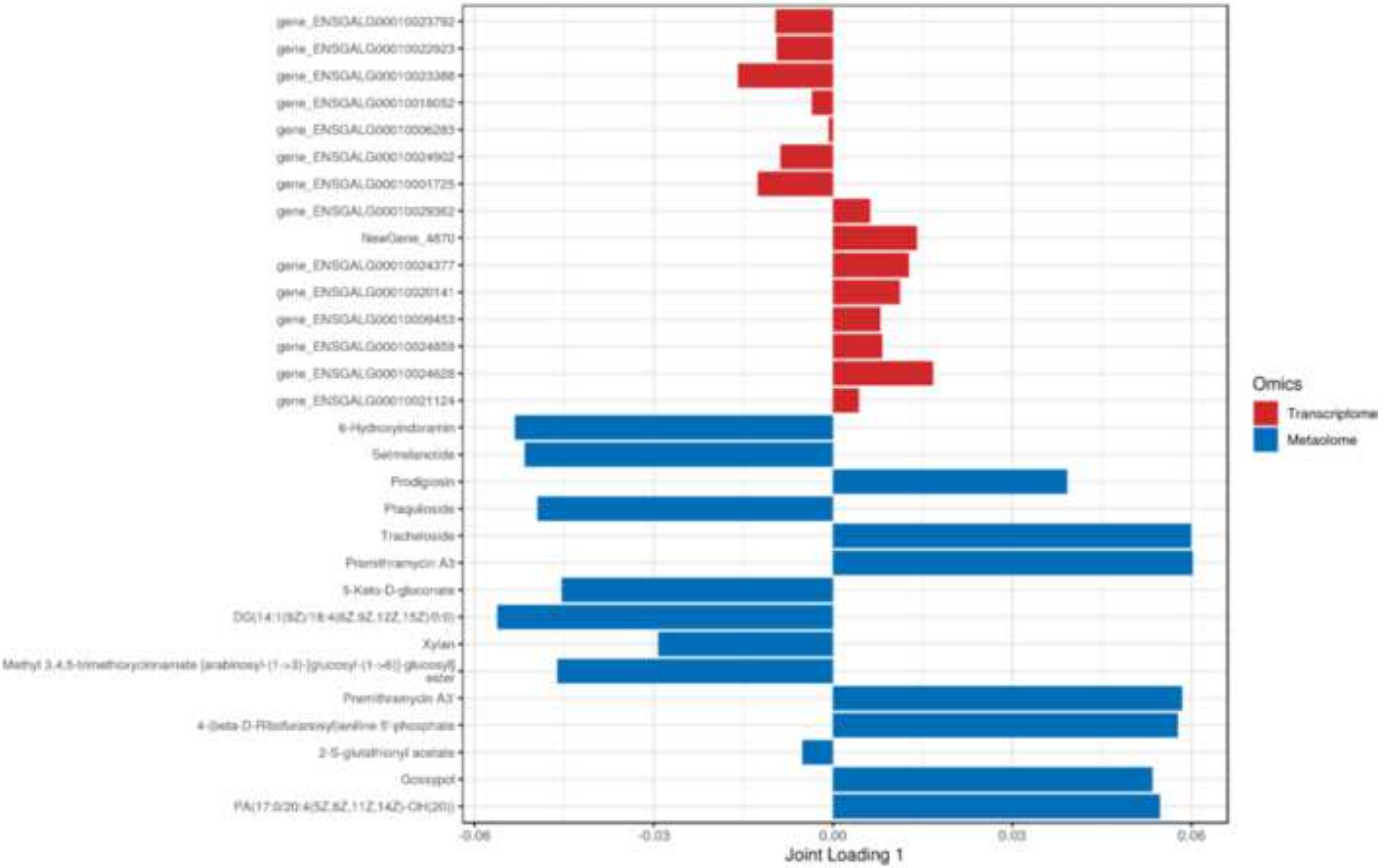

Table1. Summary statistics of sequence quality and comparison information.

Samples	Clean reads	Clean bases	GC Content	%≥Q30
ML1	20,315,207	6,078,541,552	52.83%	91.26%
ML2	21,641,148	6,474,947,590	53.05%	91.99%
ML3	23,440,977	7,011,163,862	52.42%	92.62%
ML4	19,689,063	5,890,581,038	52.93%	91.99%
ML5	21,212,590	6,345,204,430	52.77%	92.00%
MH1	22,064,781	6,598,044,174	53.38%	94.93%
MH2	22,248,260	6,656,734,114	52.36%	92.98%
MH3	22,280,888	6,665,761,316	52.64%	92.48%
MH4	21,542,599	6,445,733,214	52.96%	90.43%
MH5	20,685,551	6,187,410,710	52.37%	91.56%

(1) Samples: sample analysis number;

(2) Clean reads: Total paired-end reads post-quality filtration

(3) Clean bases: Clean Data total bases;

(4) GC content: GC composition ratio in quality-filtered sequencing data

(5) ≥Q30%: Percentage of high-quality bases (Q≥30) in processed sequencing reads

Click here to access/download;Figure;Table2_01.tif

Table2. |log2(FC)| -The top ten genes that differ

Gene	Full name	|log2(FC)|	UP/DOWN
MHM2	*male hypermethylated region 2*	3.4	UP
KCNK3	*Potassium Two Pore Domain Channel Subfamily K Member 3*	2.59	UP
ITGB6	*integrin subunit beta 6*	2.28	UP
TET2	*Ten-Eleven Translocation 2*	2.25	UP
GIPC2	*GIPC PDZ domain-containing family member 2*	2.14	UP
MYOZ2	*Myozenin 2*	2	UP
CREB5	*cAMP-responsive element-binding protein 5*	2	UP
TMEM8C	*Transmembrane Protein 8C*	1.94	UP
TRPC3	*Transient Receptor Potential Canonical 3*	2.24	DOWN
ATP6V1G3	*ATPase H+ transporting V1 subunit G3*	2.1	DOWN

Click here to access/download

Supporting Information

Vertebrate Animal Study Approval--LIU Wei.pdf
