# Supplementary material for: Integrated transcriptome and metabolome analysis of melanin-related genes and metabolites in Muchuan Black Bones Chickens
Source: PLoS One. 2025 Dec 30;20(12):e0338827. doi: 10.1371/journal.pone.0338827 (PMC12752978; doi:10.1371/journal.pone.0338827)
Supplement: Attachment — Submitted filename: Response Letter#.docx [file pone.0338827.s002.docx]

Dear Editor,

We would like to sincerely thank you and the reviewers for the time and effort you have devoted to evaluating our manuscript entitled "Transcriptome and Metabolome Combined to Analyze the Key Genes and Metabolites Related to the Melanin Content of Muchuan Black Bones Chickens" (Manuscript ID:PONE-D-25-36595). We greatly appreciate the constructive comments and valuable suggestions, which have helped us to improve the quality of our work.

We have carefully revised the manuscript according to the reviewers’ comments. In the revised version, all changes have been highlighted in red. Below, we provide a detailed point-by-point response to each comment.

1. ***The title is accurate but very long. Consider shortening it for impact.***

Response: Thank you for this helpful suggestion. We have revised the title to make it more concise while retaining the essential meaning. The new title is: Integrated Transcriptome and Metabolome Analysis of Melanin-related genes and Metabolites in Muchuan Black Bones Chickens.

2. ***In te summary section author mentions "Glucose-6-phosphate" as a key metabolite, but this compound is not mentioned again in the abstract's results section or discussed later. The results section lists different metabolites (CDP-ethanolamine, m-trigallic acid, etc.). This discrepancy must be resolved for accuracy. Ensure the abstract perfectly reflects the most significant findings presented in the paper.***

Response: Thank you for your reminder. We have removed Glucose-6-phosphate in simple summary.

3. ***However, Muchuan Black Bones Chickens exhibit segregation instead "However, its exhibit segregation***

Response: Thank you for your suggestion. We have revised the manuscript accordingly and replaced the original sentence with the suggested wording. The revised sentence now reads:”however, they exhibit segregation in melanin deposition”. This change can be found on Page 4, Line 52 of the revised manuscript.

4. ***If possible give the space between the last word and reference.***

Response: Thank you for your reminder. We have made revisions to the entire text based on your suggestions.

5. ***Previously, molecular biological methods for studying melanin deposition included gene expression analysis[13], SNP molecular marker screening[14], transcriptomics, and proteomics[15]. Instead "Previously, many molecular biological methods studying the melanin deposition included transcriptomic and proteomic [13, 14], SNP molecular marker screening[15].***

Response: Thank you for your suggestion. We have revised the manuscript accordingly and replaced the original sentence with the suggested wording. The revised sentence now reads: ”Previously, many molecular biological methods studying the melanin deposition included transcriptomic and proteomic [13-14], SNP molecular marker screening [15].”This change can be found on Page 5, Line 71-73 of the revised manuscript.

6. ***The manuscript would benefit from clearly stated research hypotheses and objectives. These should be included in the abstract (26-29) and at the end of the introduction section (e.g., around line 75-80) to improve the readability and focus of the study.***

Response: Thank you for this valuable suggestion. We have revised the manuscript accordingly by explicitly stating the research hypotheses and objectives in both the abstract (Page 2, Lines 30-32) and the introduction section (Page 5, Lines 79-81). We believe these additions improve the readability and clarity of the study.

7. ***It would be strengthened by a more precise hypothesis or set of research questions. What specifically did you expect to find by using this multi-omics approach on 35-day-old chickens?***

Response: Thank you for this insightful comment. We have revised the manuscript to clarify our research hypothesis and objectives. Specifically, we expected that applying a combined transcriptomic and metabolomic approach to 35-day-old Muchuan black-bone chickens would identify key genes and metabolites associated with early melanin deposition in breast muscle. This age was chosen because it represents a critical stage of muscle development when melanin accumulation is particularly evident. The revised text has been added to the abstract (Page 2, Lines 30-32) and the introduction section (Page 5, Lines 79-81).

8. ***If possible remove this part from here and paste in the decleration section in the end of manuscript***

Response: Thank you for your suggestion. We have made corresponding revisions to the article. The original paragraph has been moved to page 22, line 460-462.

9. ***It is stated that 60 chickens were selected. How were these 60 chosen from the larger population? Was it a random selection? This is important to avoid selection bias.***

Response: Thanks for your reminder, Sixty chickens were randomly selected. Then, the melanin content of these 60 chickens was measured. Next, the five chickens with the highest and lowest melanin levels were selected for analysis. We made corresponding modifications to the sentence by adding "randomly" (Page 5, Lines 91-92).

10. ***A total of 60 35-day-old Muchuan Black Bones Chickens were selected. instead "A total of 60 chickens with the age of 35-day old***

Response: Thanks for your reminder, We have already replaced this sentence (Page 5, Lines 91-92).

11. ***remove one full stop***

Response: Thanks for your reminder, we have made the necessary revisions here and also checked the entire text to ensure there are no similar errors and to make the necessary corrections.

12. ***Specify the exact gas and protocol used for euthanasia ("gas fainting" is not standard).***

Response: Thank you for your reminder, we have added the method of euthanasia in page 6, line 93-97.

13. ***remove space***

Response: Thanks for your reminder, we have made the necessary revisions here and also checked the entire text to ensure there are no similar errors and to make the necessary corrections.

14. ***Provide a citation or validation data for the use of an ELISA kit to measure melanin, as it is non-standard.***

Response: Thanks for your reminder,we have provided a reference for the ELISA kit in determining melanin (Reference 21).

15. ***Specify the volume of PBS used for homogenization.***

Response: Thanks for your reminder,After reviewing our experimental procedures, we have increased the volume of PBS (page 6, line 104).

16. ***What is MH,What is ML***

Response: Thanks for your reminder,MH stands for "high melanin" and represents the high melanin group, while ML stands for "low melanin" and represents the low melanin group.Based on the levels of melanin in the breast muscle.

17. ***Acknowledge that n=5 per group is acceptable but on the lower end, which is a limitation for detecting low-expression DEGs.***

Response: Thanks the reviewer for this valuable comment. While a sample size of n = 5 per group is acceptable in transcriptomic studies, we acknowledge that it represents the lower bound of statistical power and may limit the detection of lowly expressed DEGs. This point has been clarified in the revised manuscript (page 7, line 117-119).

18. ***is mRNA or sRNA?***

Response: Thanks for your reminder, we confirmed that it was mRNA by reviewing the raw data.

19. ***The word gurantee replace with confirm. confirm is not a sceitific word. Please correct this type of mistakes in whole manuscript.***

Response: Thanks for your reminder, we conducted a full-text search and made the necessary modifications. The modified words have been highlighted in red.

20. ***Report the range of RIN values.***

Response: Thanks for your reminder,we have supplemented the RIN value. The range of the integrity evaluation indicator RIN is 7 - 7.6 (page 7, line 123).

21. ***Use RT-qPCR***

Response: Thanks for your reminder, we made the necessary modifications (page 8, line 143).

22. ***The primer sequences must be provided in a supplementary table.***

Response: Thanks for your reminder, we have supplemented the primer sequences, as detailed in Table 1.

23. ***write abbreviation like "qPCR"***

Response: Thanks for your reminder, we have made the necessary changes here (page 8, line 147).

24. ***delete space***

Response: Thanks for your reminder, we have made the necessary changes here (page 8, line 148).

25. ***the standard calculation method is 2-delta delta CT, not 2 delta delta CT***

Response: Thanks the reviewer for noting this error. The formula was mistakenly shown as 2^∆∆^Ct due to a formatting issue. It has been corrected to 2−^∆∆^Ct in the revised manuscript. The analysis itself was performed using the standard 2−^∆∆^Ct method, so the results and conclusions remain unaffected (page 8, line 149).

26. ***Specify the exact composition of the "extraction solvent" (e.g., methanol:water, 80:20).***

Response: Thanks for your reminder, the extractant composition has been clearly defined and has been revised in the article (page 8-9, line 158-159).

27. ***delete one full stop***

Response: Thanks for your reminder, we conducted a full-text search and made the necessary modifications.

28. ***Also describe the ionization mode(s) (Positive/Negative) and mass scan range.***

Response: Thanks for your reminder, we have already made up for this part (page 9, line 163-170).

29. ***Because in the result section 3.4.1 author write WGCNA analysis so the methodology should be present in the method section.Where is the WGCNA analysis methodology WGCNA***

Response: Thanks for your reminder, we have already made up for this part (page 10, line196-201).

30. ***If it possibe cite the figure in the last of text or should present like "Figure 1 C as illustrated in the volcano plot.***

Response: Thanks for your suggestion, we made adjustments to the sentence and made corresponding changes in the text (page 11, line 204-206). And the incorrect word order of other figures has also been appropriately modified (page 12, line 227) (page 14, line 280, line 288-289) (page 15, line 298-299, line 305, line 309).

31. ***Where is the Q20 value?***

Response: Thanks for your reminder, we reviewed the original data, found the corresponding data, and made the necessary modifications in Table 3 and in the article page 11, line 217-218).

32. ***The P value mentioned accurately in the same way if you want to presented in italic then correct in entire the manucript.***

Response: Thanks for your reminder, we conducted a full-text search and made the necessary modifications. The modified words have been highlighted in red.

33. ***The results for pathway enrichment are presented but not interpreted. For example:"Integrin binding" (Line 216): Propose a link to melanocyte migration/adhesion."Neuroactive ligand-receptor interaction" (Line 220): Explicitly state this makes sense as melanogenesis pathways (e.g., MC1R ) are neuroendocrine.***

Response: Thanks for your reminder, we have made the necessary revisions at the corresponding positions (page 13, line 249-250; line 251-253).

34. ***The connection between the identified pathways (e.g., Neuroactive ligand-receptor interaction, Arginine-proline metabolism) and melanogenesis is not immediately obvious. The discussion should more explicitly hypothesize the mechanistic links***

Response: Thanks for your reminder, in this section, we have made additional remarks in the discussion (page 18 line 375-381).

35. ***Show the expression heat map and also discuss the expression value of gene***

Response: Thanks for your reminder，the gene expression situation has been included as an appendix, so the heatmap will no longer be provided (Table 5), and the article has been appropriately revised (page 13, line 255-263).

36. ***The text merely lists metabolites. Group them by class/function here for greater impact (e.g., "compounds involved in phospholipid synthesis (CDP-ethanolamine)").***

Response: Thanks for your suggestion, we classified and organized them according to their functions (page 14, line 281-285).

37. ***please describe all results briefly not in the 2 or 3 lines***

Response: Thanks for your suggestion, we have condensed this text (page 14-15, line 287-293).

38. ***The biological meaning of "Histidine metabolism" and "Terpenoid backbone biosynthesis" is not discussed. Are they related to antioxidant capacity or melanosome membranes?***

Response: Thanks for your suggestion, we have appropriately supplemented the biological significance of the "Histidine metabolism" and "Terpenoid backbone biosynthesis" pathways (page 14, line 289-293).

39. ***This section is descriptive but lacks a conclusive description for instance; The greatest potential lies in direct integration. Perform and present a correlation analysis between top DEGs (e.g., CREB5, GIPC2) and top metabolites to generate powerful hypotheses (e.g., Is CREB5 expression correlated with UDP-glucuronic acid levels?).***

Response: Thanks for your suggestion, it has been modified in the text (page 15, line 306-315).

40. ***The discussion on CREB5 and the cAMP-PKA-MITF pathway is excellent. The biological significance of the other enriched pathways (e.g., arginine-proline metabolism, histidine metabolism) from the results is underdeveloped.***

Response: Thanks for your suggestion, We have appropriately supplemented the biological significance of arginine-proline metabolism and histidine metabolism (page 17-18, line 360-375).

41. ***For CDP-ethanolamine, elaborate on how membrane fluidity might affect melanosome biogenesis.***

Response: Thanks for your reminder, we have conducted supplementary research on how membrane fluidity affects the formation of melanosome structures (page 20-21, line 418-424).

42. ***The abbreviation should be PC not PE, correct this also and also entire the manuscript.***

Response: Thanks for your reminder, after a careful examination, we found that it was not an error in the abbreviation, but rather that "phosphatidylethanolamine" was mistakenly written as "phosphatidylcholine". Although both PC and PE are important substances for the synthesis of membrane structures, CDP-ethanolamine is the substance used for the synthesis of PE. We have made the correction in the article (page 20, line 418).

43. ***The explanation for not detecting classic genes like TYR or MITF is reasonable. Frame this as a strength: the approach uncovered novel regulators beyond the core pathway.***

Response: Thanks for your reminder, we are grateful for the insightful suggestion made by the reviewer. Following the reviewer's advice, we have supplemented the discussion here, emphasizing that our method has identified new regulatory factors beyond the core pathway (page 22, line 448-450).

44. ***The conclusion is too short. The conclusion is also concise and would benefit from a more detailed summary of the key findings. Moreover, the authors should suggest future research directions or potential applications based on their results.***

Response: Thanks for your reminder, we have summarized the key findings of the conclusion in detail, and based on the research results, we have identified the potential value of future research (page 21-22, line 452-458).
