# Supplementary material for: Integrated transcriptome and metabolome analysis of melanin-related genes and metabolites in Muchuan Black Bones Chickens
Source: PLoS One. 2025 Dec 30;20(12):e0338827. doi: 10.1371/journal.pone.0338827 (PMC12752978; doi:10.1371/journal.pone.0338827)
Supplement: Attachment — Submitted filename: Response Letter-2.docx [file pone.0338827.s003.docx]

Dear Editor,

We would like to sincerely thank you and the reviewers for the time and effort you have devoted to evaluating our manuscript entitled "Transcriptome and Metabolome Combined to Analyze the Key Genes and Metabolites Related to the Melanin Content of Muchuan Black Bones Chickens" (Manuscript ID:PONE-D-25-36595). We greatly appreciate the constructive comments and valuable suggestions, which have helped us to improve the quality of our work. We have carefully revised the manuscript according to the reviewers’ comments. Below, we provide a detailed point-by-point response to each comment.

***Journal Requirements:***

***Please review your reference list to ensure that it is complete and correct. If you have cited papers that have been retracted, please include the rationale for doing so in the manuscript text, or remove these references and replace them with relevant current references. Any changes to the reference list should be mentioned in the rebuttal letter that accompanies your revised manuscript. If you need to cite a retracted article, indicate the article’s retracted status in the References list and also include a citation and full reference for the retraction notice.***

Response:We reviewed all cited references using the Retraction Watch Database, and no retracted publications were identified.

***The manuscript presents a well-designed multi-omics study that "Integrated Transcriptome and Metabolome Analysis of Melanin-related genes and Metabolites in Muchuan Black Bones Chickens". The identification of novel candidate genes, and metabolites is a valuable contribution to the field. The revisions made in response to the previous editorial and reviewer comments have significantly improved the manuscript's clarity and depth.***

***However, several issues, primarily concerning language, clarity, and data interpretation, still need to be addressed before the manuscript is suitable for publication.***

Response:We made revisions to the manuscript, improving the language expression, enhancing the clarity of the charts, and modifying the image sizes, in an effort to meet the requirements of the journal. However, changing the size of the image may affect some clarity.
